# Impacts of updated reaction kinetics on the global GEOS-Chem simulation of atmospheric chemistry

**DOI:** 10.5194/gmd-17-1511-2024

**Published:** 2024-02-20

**Authors:** Kelvin H. Bates, Mathew J. Evans, Barron H. Henderson, Daniel J. Jacob

**Affiliations:** 1 School of Engineering and Applied Sciences, Harvard University, Cambridge, MA 02138, USA; 2 NOAA Chemical Sciences Laboratory, Earth System Research Laboratories, and Cooperative Institute for Research in Environmental Sciences, University of Colorado, Boulder, CO 80305, USA; 3 Wolfson Atmospheric Chemistry Laboratories, Department of Chemistry, University of York, York, UK; 4 National Centre for Atmospheric Science, University of York, York, UK; 5 U.S. Environmental Protection Agency, Research Triangle Park, NC 27711, USA

## Abstract

We updated the chemical mechanism of the GEOS-Chem global 3-D model of atmospheric chemistry to include new recommendations from the NASA Jet Propulsion Laboratory (JPL) chemical kinetics Data Evaluation 19–5 and from the International Union of Pure and Applied Chemistry (IUPAC) and to balance carbon and nitrogen. We examined the impact of these updates on the GEOS-Chem version 14.0.1 simulation. Notable changes include 11 updates to reactions of reactive nitrogen species, resulting in a 7% net increase in the stratospheric NO_*x*_ (NO + NO_2_) burden; an updated CO + OH rate formula leading to a 2.7% reduction in total tropospheric CO; adjustments to the rate coefficient and branching ratios of propane + OH, leading to reduced tropospheric propane (−17%) and increased acetone (+3.5%) burdens; a 41% increase in the tropospheric burden of peroxyacetic acid due to a decrease in the rate coefficient for its reaction with OH, further contributing to reductions in peroxyacetyl nitrate (PAN; −3.8%) and acetic acid (−3.4%); and a number of minor adjustments to halogen radical cycling. Changes to the global tropospheric burdens of other species include −0.7% for ozone, +0.3% for OH (−0.4% for methane lifetime against oxidation by tropospheric OH), +0.8% for formaldehyde, and −1.7% for NO_*x*_. The updated mechanism reflects the current state of the science, including complex chemical dependencies of key atmospheric species on temperature, pressure, and concentrations of other compounds. The improved conservation of carbon and nitrogen will facilitate future studies of their overall atmospheric budgets.

## 1 Introduction

Chemical reactions underpin the trace gas composition of the atmosphere, removing some pollutants and climate-forcing species while forming others. Accurate representation of reaction rate coefficients and product distributions of these reactions is crucial for atmospheric chemistry models. The NASA Jet Propulsion Laboratory (JPL) regularly assembles a panel of experts to assess and compile new data for rate constants and other key reaction parameters relevant to atmospheric chemistry. This panel produces data evaluations entitled “Chemical Kinetics and Photochemical Data for Use in Atmospheric Studies”. Recommendations are predominantly based on laboratory measurements, although they may also be informed by computational studies or by analogy to similar reactions when required and are not adjusted to fit ambient observations. Where assessed experimental studies disagree on reaction parameters, the panel attempts to reconcile and/or arbitrate differences and provide estimated uncertainty bounds. A parallel evaluation process is conducted by the International Union of Pure and Applied Chemistry (IUPAC) with more emphasis on organic chemistry (Atkinson et al., 2006).

Here, we update the chemical mechanism in the GEOS-Chem global atmospheric chemistry model with recommendations from the latest JPL Data Evaluation (Burkholder et al., 2020) and recent IUPAC updates (Mellouki et al., 2021). The GEOS-Chem model is used by hundreds of research groups worldwide for global and regional studies of tropospheric and stratospheric chemistry (http://geos-chem.org, last access: 16 February 2024) and is also used as a chemistry module in meteorological and climate models (Hu et al., 2018; Lin et al., 2021; Fritz et al., 2022). We enforce carbon and nitrogen conservation in a further 63 reactions to facilitate budget analyses (Safieddine et al., 2017). We examine the effects of these different changes on the GEOS-Chem simulation as documentation for their inclusion in the standard version of the model.

## 2 GEOS-Chem chemical mechanism updates

The GEOS-Chem model includes a detailed mechanism for oxidant–aerosol chemistry in the troposphere and stratosphere. Since its first iteration as a model of tropospheric oxidant chemistry (Bey et al., 2001), the standard GEOS-Chem chemical mechanism has used kinetics and products based on JPL recommendations (DeMore et al., 1997). The model was later expanded to include aerosol chemistry (Park et al., 2004) and stratospheric chemistry (Eastham et al., 2014), and reaction rates and products were updated based on subsequent JPL data evaluations (Sander et al., 2006, 2011; Burkholder et al., 2015). Photolysis frequencies are calculated with the Fast-JX code (Bian and Prather, 2002) as implemented in GEOS-Chem by Mao et al. (2010) for the troposphere and by Eastham et al. (2014) for the stratosphere. Subsequent additions to the mechanism, such as detailed halogen chemistry (initially from Sherwen et al., 2016, and Chen et al., 2017, subsequently updated by Wang et al., 2021) and schemes for the oxidation of isoprene (initially from Mao et al., 2013, updated by Marais et al., 2016, and Bates and Jacob, 2019), monoterpenes (Fisher et al., 2016), and aromatics (Bates et al., 2021), include reactions not assessed in the JPL data evaluations; kinetics and products of these reactions use recommendations from IUPAC (Atkinson et al., 2006) or other sources detailed in the publications describing each update. More information on the GEOS-Chem mechanism can be found in the model documentation at http://www.geos-chem.org (last access: 16 February 2024).

Here we focus on gas-phase thermal chemistry updates as given by JPL and IUPAC. Rate coefficients from the latest JPL recommendations are prioritized over those from IUPAC, the latter of which are only implemented for reactions of oxygenated organics not included in the JPL Data Evaluation. We do not include photolysis updates, as these are not significantly changed in the latest JPL publication for reactions included in GEOS-Chem, or aerosol and cloud chemistry, for which current GEOS-Chem treatments are documented in Holmes et al. (2019), Shah et al. (2020), and Wang et al. (2021). A total of 63 gas-phase thermal reactions (out of the 650 in GEOS-Chem version 14.0.1, in addition to 157 photolyses and 107 aerosol and cloud reactions) are updated with new rate coefficients and/or product distributions, including one newly added reaction. Table 1 lists the reactions updated with the old and the new rate coefficients and products given, and Figs. S1–S9 in the Supplement show the impact of these updates on rate coefficients as a function of temperature and/or pressure. The following paragraphs provide a summary of the changed rate coefficients and product yields, organized by chemical family as in Table 1. All updates are from the JPL Data Evaluation unless otherwise noted.

A total of 11 updated reaction rate coefficients correspond to inorganic reactions in the reactive nitrogen (NO_*y*_ = NO + NO_2_ + all oxidized odd nitrogen species) family, with new values derived predominantly from changes to the methods of combining data from previous studies. Most notable among these updates is a reversal in the temperature dependence from positive to negative for the reaction between nitrous acid (HONO) and OH, resulting in a 40% increase in the rate coefficient at 300K rising to a 300% increase at 200K. The new assessment prioritizes the temperature dependence measured by Burkholder et al. (1992) over that of Jenkin and Cox (1987), which had previously been used. Rate coefficient formulas for the two pressure- and temperature-dependent branches of the O + NO_2_ reaction (association to yield NO_3_ and activation to yield NO + O_2_) were rebalanced, resulting in increases to the former branch of 19%–21% and decreases to the latter of 10%–18% (producing an overall decrease of 6%–9%) under typical conditions between altitudes of 0km (288K; 1013 hPa) and 10 km (240K; 270 hPa). The combined termolecular activation and association reactions of OH + HNO_3_, both yielding NO_3_ + H_2_O, were also reanalyzed based on new experimental work by Winiberg et al. (2018) and Dulitz et al. (2018), resulting in changes to the rate coefficient of −1% to +4% over the range of atmospheric conditions. Other updates in this family include a rate coefficient increase for NO_3_ + O (+30%) and decreases for OH + NO_3_ (−9%) and NO_2_ + NO_3_ (−33% to −25% over the range 200–300K); weaker temperature dependencies and adjusted rate constants for HO_2_ + NO, NO + NO_3_, and N + O_2_, resulting in changes to their coefficients over the range 200–300K of −1% to +1%, −10% to −2%, and +109% to −1%, respectively; and a stronger temperature dependence for OH + HNO_4_ (+9% to −25%).

Of these changes, 13 are updates to branching ratios in reactions of O^1^D with HBr, HCl, and halogenated organic compounds. These adjustments are generally minor, shifting the branching between the O + *XR* and O*X* + *R* product channels (where *X* stands in for Cl or Br and *R* stands in for H or an organic group) by a few percent. We also add the newly included O^1^D + CH_3_Cl reaction and update the temperature dependencies of the O^1^D + CFC114 and O^1^D + CFC115 reactions to the JPL recommendations based on Baasandorj et al. (2013), resulting in increases of between 18% and 35% to their rate coefficients over the range 200–300K.

A total of four updates apply to the rate coefficients of organic ozonolysis reactions. While three of these (O_3_ + ethene, propene, and methacrolein) are only small adjustments to temperature dependence, resulting in rate coefficient increases of 2%–12% over the range 200–300K, the isoprene ozonolysis reaction in GEOS-Chem did not previously have a temperature dependence. We include that now per JPL recommendation, which considerably slows ozonolysis at low temperatures (−96% at 200K and −44% at 273K) and accelerates it when *T* > 297K.

A total of 10 rate coefficients of OH + organic reactions are changed, with five based on JPL recommendations and five from IUPAC. From JPL, the notable update is to the two C_3_H_8_ + OH branches (primary and secondary abstraction). The two branches have different temperature dependencies, a feature which had previously been included in GEOS-Chem, but the reanalysis in this JPL Data Evaluation changes both dependencies considerably, reversing the dependence of the secondary abstraction. Overall, the importance of the secondary abstraction branch increases: its rate coefficient goes up 300% at 200K but barely changes at 300K, while the primary abstraction branch goes up 140% at 200K but drops 30% at 300K. This is after accounting for the apparent typo in the primary abstraction rate constant from the JPL Data Evaluation, which would increase its rate coefficient by a factor of 10. The other JPL-derived revisions include a reduction in the OH oxidation rate coefficient for C_3+_ alcohols (“ROH”) by 4% and weaker temperature dependencies for OH + CH_2_Cl_2_ and CH_3_Cl, changing their rate coefficients by +2% to −9% and by +37% to −3%, respectively, over the temperature range 200–300K. The IUPAC-derived updates include the addition of a temperature dependence for the methylglyoxal + OH reaction based on Baeza-Romero et al. (2007) and Tyndall et al. (1995), changing its rate coefficient by +125% at 200K and −14% at 300K; minor changes to the temperature dependencies of the methyl ethyl ketone + OH rate coefficient (−17% to −7% at 200–300K) and to both branches of the hydroxyacetone + OH reaction (0% to −2%); and a factor of 30–50 downward revision to the OH + peroxyacetic acid (PAA) rate coefficient based on the experimental and theoretical results of Berasategui et al. (2020).

A total of five rate coefficients of reactions between the Cl radical and organic compounds are updated. The Cl + acetone rate coefficient now has a smaller temperature dependence and faster rate at temperatures below 251K, resulting in a slower reaction by 22% at 300K but a faster reaction by 49% at 200K than previously. The Cl + CH_2_O reaction has the same temperature dependence, but its rate constant is revised upward by 11%. The other three changes are to Cl + chloromethane compounds: the Cl + CH_2_Cl_2_ rate constant, though unchanged in Publication 19–5 from previous JPL data evaluations, may have been erroneously low in GEOS-Chem because we find it requires increasing by a factor of 10.2–13.3 over the range 200–300K to match the recommendation. The updates to the Cl + CH_3_Cl and Cl + CHCl_3_ coefficients are much smaller, changing by +3%–0% and −3% to −6%, respectively, over the range 200–300K.

A total of five revisions apply to reactions of organic peroxy (RO_2_) radicals, including increases in the rate coefficients of the self-reaction of the peroxyacetyl radical (CH_3_CO_3_) by 14%, the reaction of NO with the acetone-derived peroxy radical CH_3_C(O)CH_2_O_2_ by 4%, the reaction of the ethylperoxy radical (CH_3_CH_2_O_2_) with HO_2_ (+1%), and other functionalized C_2_ peroxy radicals + HO_2_ (+1%). These updates also include a slight increase in the temperature- and pressure-dependent peroxyacetyl nitrate (PAN) formation rate (the termolecular reaction of CH_3_CO_3_ + NO_2_) by about 2% under typical conditions between altitudes of 0km (288K; 1013 hPa) and 10km (240K; 270 hPa). However, the PAN equilibrium remains unchanged in this JPL assessment, so we also adjust the dissociation reaction rate coefficient accordingly.

A total of four updates pertain to the fates of Criegee intermediates. Some of these reactions are newly included in the JPL Data Evaluation and had previously received scant attention in GEOS-Chem: they were generally added selectively to the mechanism either to provide a source of HCOOH (Millet et al., 2015) or to complete the isoprene oxidation cascade (Bates and Jacob, 2019). Updates include substantial increases in the rate coefficients of CH_2_OO + NO_2_ (+4250%) and CH_3_CHOO + SO_2_ (+377%), a slight increase to CH_2_OO + SO_2_ (+3%), and a sharp decrease to the rate coefficient of CH_2_OO + H_2_O (−84%).

A total of six changes are made to reactions in the iodine radical chemistry scheme, most notably a downward revision by 54% to the IO + ClO rate constant. The temperature dependence of the IO + BrO reaction is increased, resulting in changes to its rate coefficient of +28% at 200K ranging to −16% at 300K. Conversely, temperature dependencies are weakened for the IO + NO and I + O_3_ reactions, resulting in changes of +6% to −1% and −10% to −9%, respectively, over the range 200–300K. Slight adjustments to the I + NO and I + NO_2_ rate coefficients result in decreases of <1% and <3%, respectively.

The list is completed by five other miscellaneous rate coefficient changes. First, a revision to the formula for the termolecular reaction of SO_2_ with OH results in pressure- and temperature-dependent rate coefficient decreases of 0%–5% under typical conditions between altitudes of 0km (288K; 1013 hPa) and 10km (240K; 270 hPa). Similar revisions to the formula for the termolecular H + O_2_ reaction and the combined activation and association reactions of OH + CO result in rate coefficient increases under the same range of atmospheric conditions of 23%–34% and 3%–5%, respectively. Finally, a weaker temperature dependence for the reaction of OCS with OH renders the rate coefficient nearly unchanged at 300K but 10% faster at 250K and 25% faster at 200K.

The product distributions of a further 63 reactions listed in Table S2 were adjusted so as to balance carbon and nitrogen between the reactants and products. Previously, CO_2_ had not explicitly been represented as a product in many reactions, while others were imbalanced due to rounding errors or uncertainties in product branching ratios. The updated reactions were balanced predominantly by adding CO_2_ as a coproduct, adjusting branching ratios slightly to offset rounding errors or adding lumped organic products (e.g., “RCHO” for C_3+_ aldehydes, which carries three carbon atoms) to account for products for which the specific structure is unknown. There remain 54 reactions (Table S3) that are imperfectly carbonor nitrogen-balanced: most are (a) in the monoterpene oxidation submechanism (Fisher et al., 2016); (b) reactions of (H)CFCs, which play a minor role in the atmospheric carbon budget; and (c) reactions coupling the gas-phase organic mechanism to the formation of non-specific particle-phase species (e.g., a single aerosol-phase isoprene-derived nitrate species carries five carbons and one nitrogen but is formed via uptake of species with four to five carbons and one to two nitrogens). Balancing these reactions will be the focus of future efforts.

## 3 Model simulations

We use version 14.0.1 of GEOS-Chem Classic (https://doi.org/10.5281/zenodo.7271974, The International GEOS-Chem User Community, 2022) driven by NASA-MERRA-2 assimilated meteorological data to simulate the impacts of the updated rate coefficients on trace gas concentrations and budgets. Detailed gas and aerosol chemistry is computed throughout the troposphere and stratosphere at 30 min time intervals using a fourth-order Rosenbrock kinetic solver implemented with the Kinetic PreProcessor (KPP) version 3.0 (Lin et al., 2023). Emissions are calculated at 30min time steps using the Harmonized Emissions Component (HEMCO) version 3.0 (Lin et al., 2021). This includes anthropogenic emissions from the Community Emissions Data System (CEDS) inventory (Hoesly et al., 2018); biomass burning emissions from the Global Fire Emissions Database (GFED) version 4 (van der Werf et al., 2017); and biogenic VOC emissions from the Model of Emissions of Gases and Aerosols from Nature (MEGAN) version 2.1 (Guenther et al., 2012), as implemented by Hu et al. (2015) and calculated offline to improve reproducibility across scales (Weng et al., 2020). Additional emission sources, including air–sea exchange of organics, NO_*x*_ (NO + NO_2_) from soils and lightning, and others, follow the default GEOS-Chem version 14.0 settings. Methane is treated as an advected and reactive species but without emissions; instead, surface concentrations are prescribed based on measured monthly means (Murray, 2016).

To calculate the changes in burdens and budgets of atmospheric species described in the following section, we perform two simulations: one with the unchanged chemical mechanism from GEOS-Chem classic version 14.0.1 (“base”) and another with the mechanism altered to include the updates listed in Tables 1, S2, and S4 in the Supplement (“updated”). Both simulations are performed at 2°×2.5° horizontal resolution over 72 vertical levels, are initialized from the same generic concentration fields that reflect realistic atmospheric concentrations of model species, and are run for 2 years (1 January 2017 to 1 January 2019). The first year is to remove the effect of the common initialization. We use output from the second year to compute the changes described below. While this spinup is sufficient to demonstrate direct changes to most species concentrations from updated rate coefficients and product yields, it does not reflect effects of longer-term processes like stratosphere–troposphere exchange.

## 4 Impacts on species concentrations

**Figure 1.**
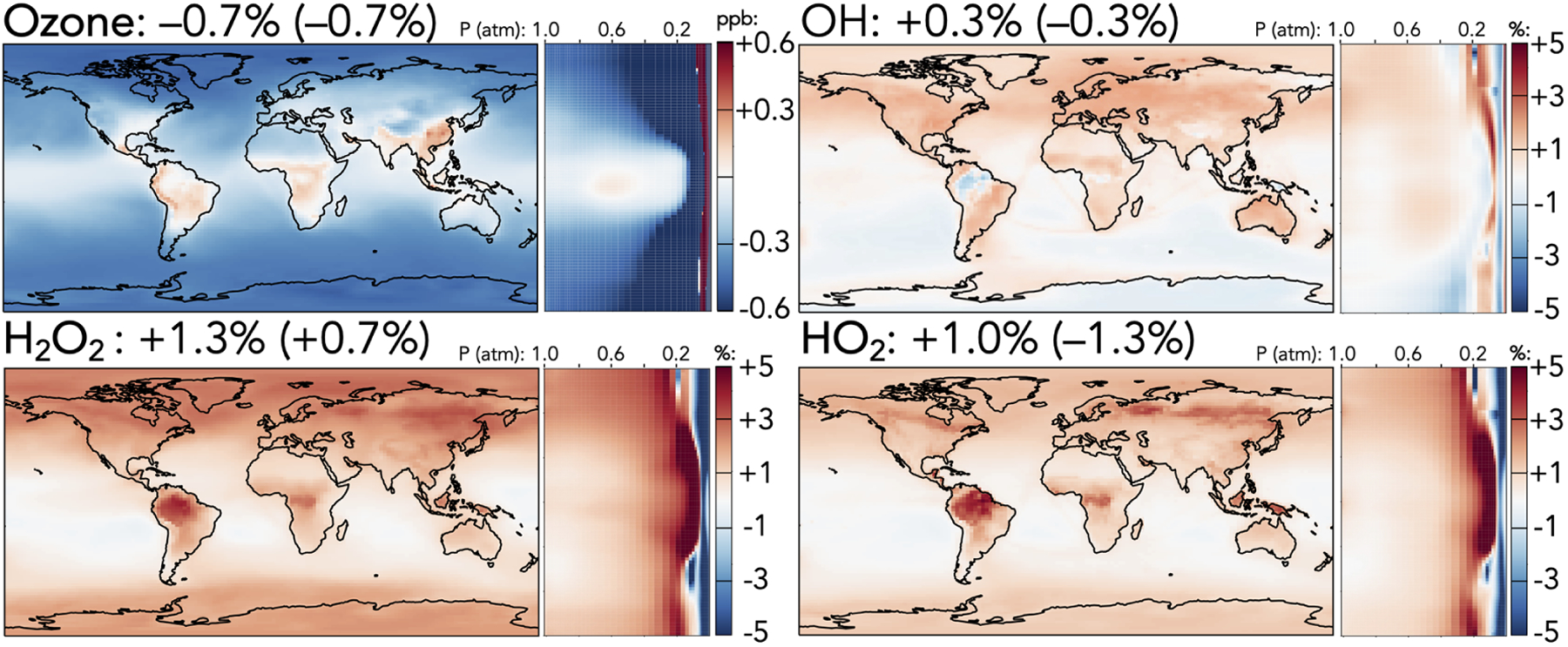
Absolute (top left, for ozone) and relative (all others) changes in the annual average mixing ratios of (H)O_*y*_ species between the base and the updated mechanism. Maps show surface values; atmospheric cross-sections show zonal means using the labeled altitude scale and the same latitude scales as the maps to their left. Additional vertical profiles of these species’ changes can be found in Fig. S12 in the Supplement. Scales differ between species but are the same for each individual species’ surface maps and cross-sections. Numbers next to species’ names show the percent change in their annual average tropospheric burden (stratospheric burden in parentheses) from the base to the updated mechanism.

**Figure 2.**
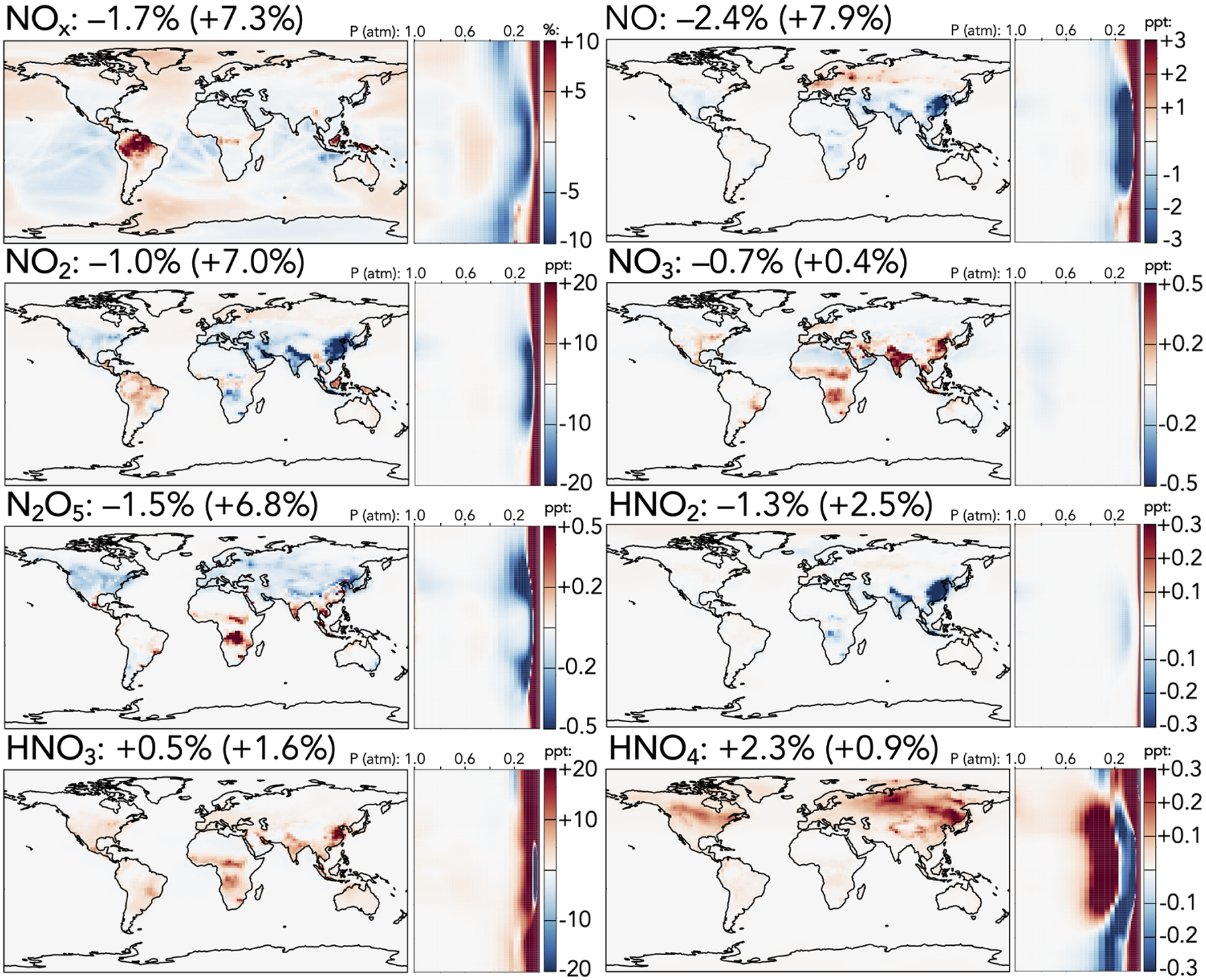
Absolute (top left, for NO_*x*_) and relative (all others) changes in the annual average mixing ratios of NO_*y*_ species between the base and the updated mechanism. Maps show surface values; atmospheric cross-sections show zonal means using the labeled altitude scale and the same latitude scales as the maps to their left. Additional vertical profiles of these species’ changes can be found in Fig. S13 in the Supplement. Scales differ between species but are the same for each individual species’ surface maps and cross-sections. Numbers next to species’ names show the percent change in their annual average tropospheric burden (stratospheric burden in parentheses) from the base to the updated mechanism.

**Figure 3.**
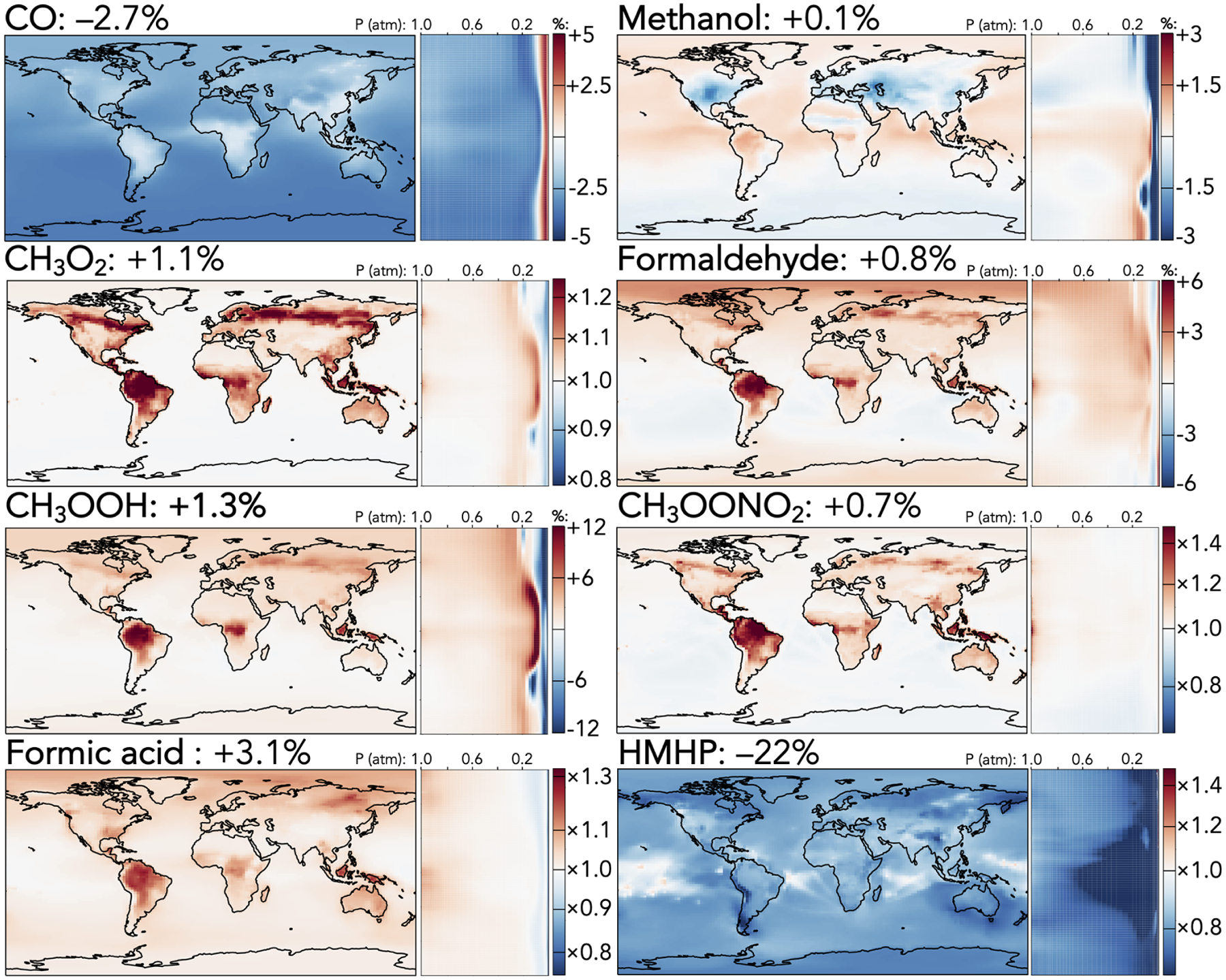
Relative changes in the annual average mixing ratios of C_1_ species between the base and the updated mechanism. Maps show surface values; atmospheric cross-sections show zonal means using the labeled altitude scale and the same latitude scales as the maps to their left. Additional vertical profiles of these species’ changes can be found in Fig. S14 in the Supplement. Scales differ between species but are the same for each individual species’ surface maps and cross-sections. Numbers next to species’ names show the percent change in their annual average tropospheric burden from the base to the updated mechanism.

**Figure 4.**
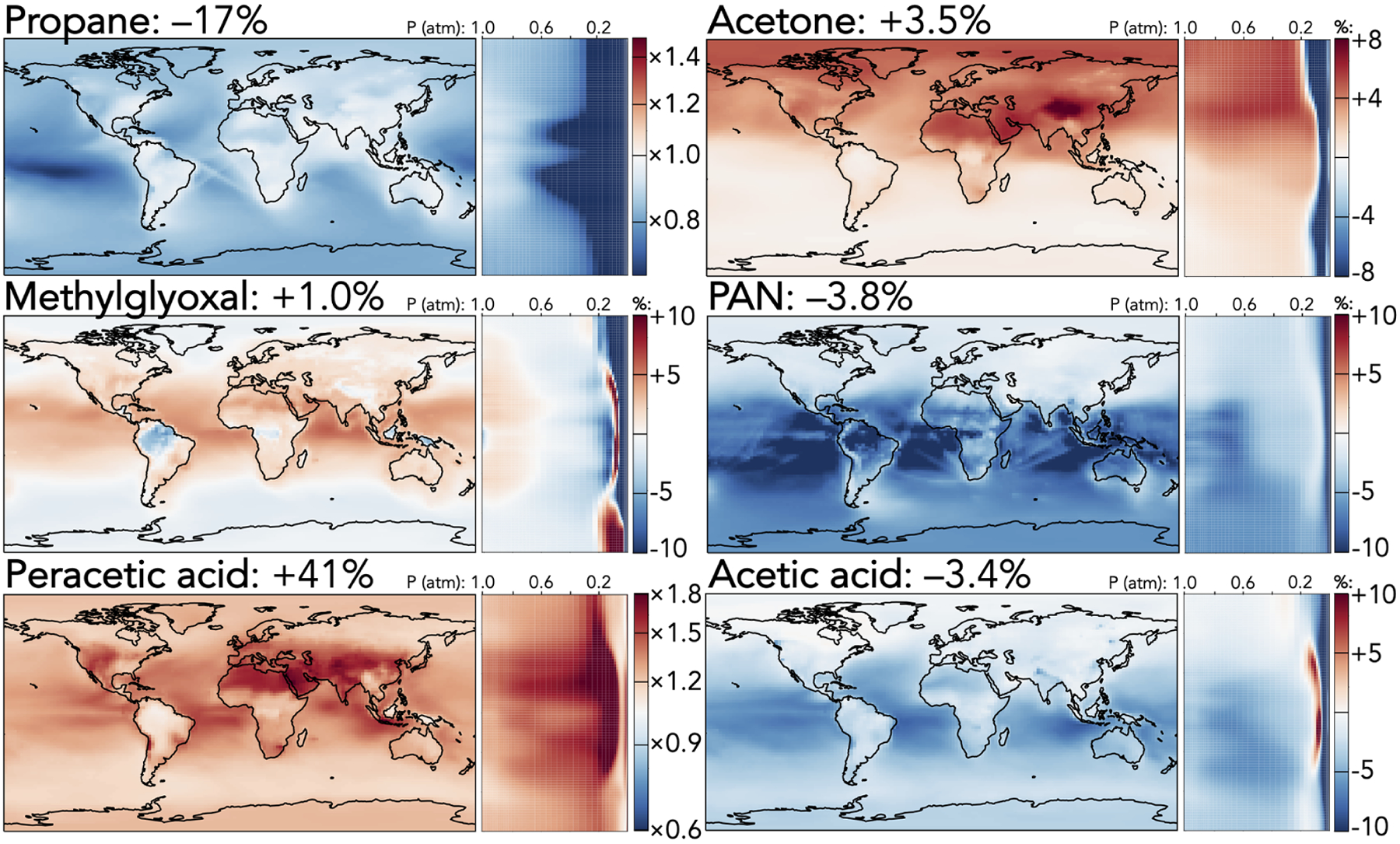
Relative changes in the annual average mixing ratios of select organic species between the base and the updated mechanism. Maps show surface values; atmospheric cross-sections show zonal means using the labeled altitude scale and the same latitude scales as the maps to their left. Scales differ between species but are the same for each individual species’ surface maps and cross-sections. Numbers next to species’ names show the percent change in their annual average tropospheric burden from the base to the updated mechanism.

**Figure 5.**
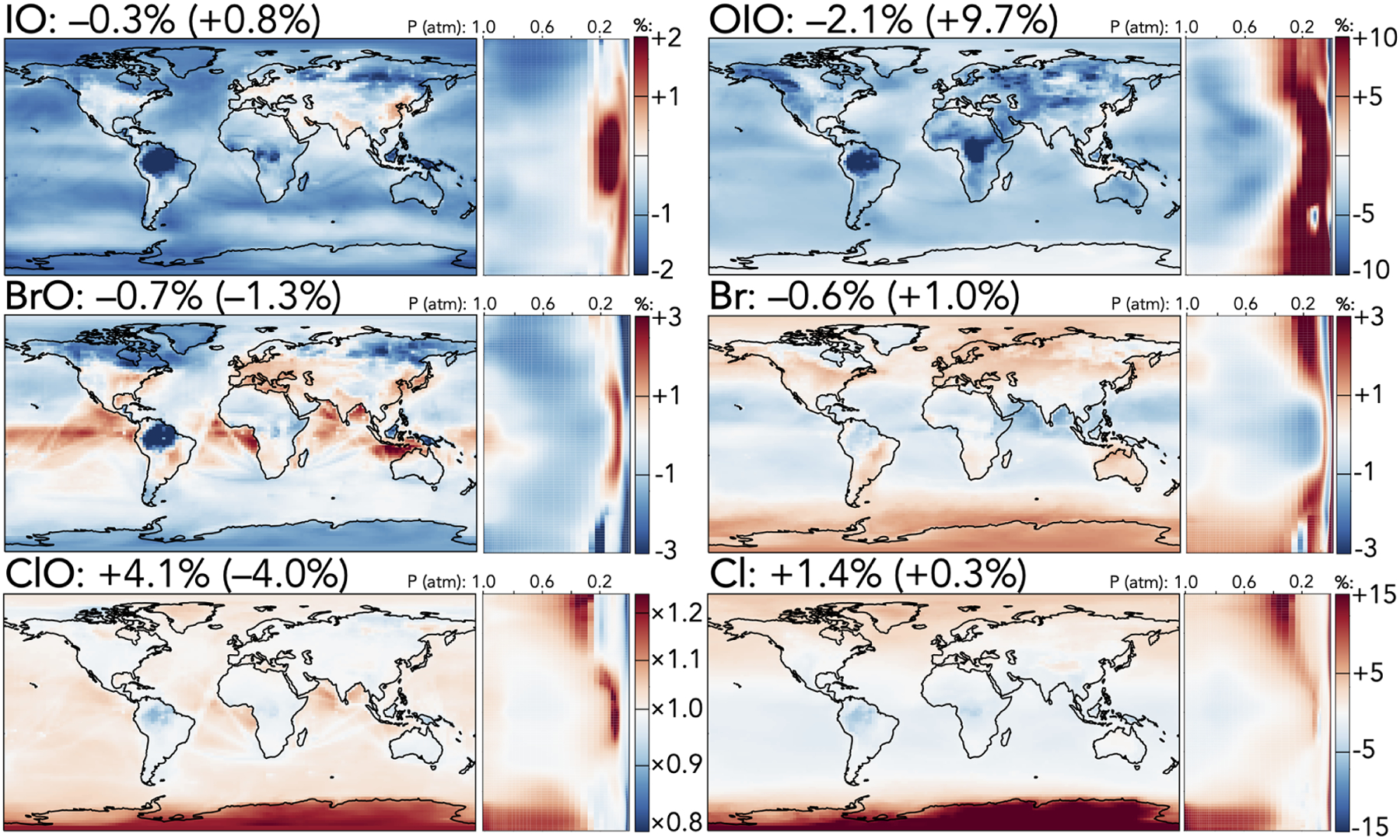
Relative changes in the annual average mixing ratios of select halogen species between the base and the updated mechanism. Maps show surface values; atmospheric cross-sections show zonal means using the labeled altitude scale and the same latitude scales as the maps to their left. Additional vertical profiles of these species’ changes can be found in Fig. S15 in the Supplement. Scales differ between species but are the same for each individual species’ surface maps and cross-sections. Numbers next to species’ names show the percent change in their annual average tropospheric burden (stratospheric burden in parentheses) from the base to the updated mechanism.

Figures 1–5 show the impacts of the rate coefficient and product yield updates on annually averaged surface mixing ratios, zonally averaged mixing ratios, and total tropospheric and stratospheric burdens of selected species. These changes are also described and explained in the following paragraphs. A more complete list of impacts on annually averaged tropospheric mass burdens for all species in GEOS-Chem that change by >1% can be found in Table S5, and a similar list for stratospheric changes can be found in Table S6.

Figure 1 shows the effects of the mechanism changes on ozone, HO_*x*_ (= OH + HO_2_), and H_2_O_2_. Total tropospheric ozone decreases by 0.7% in the updated mechanism, corresponding to a surface decrease of 0.5 ppb in the extratropics, driven in part by reduced PAN-driven NO_*x*_ transport (described below) and in part by faster organic ozonolysis reactions. Over East Asia, where faster CO + OH and C_3_H_8_ + OH reactions increase radical cycling, and over tropical forests, where isoprene ozonolysis is slowed and NO_*x*_ is increased due to better nitrogen conservation in the reaction forming methyl vinyl ketone hydroxynitrate (MVKN), surface ozone rises by 0.1–0.3ppb. The upper troposphere and mid-stratosphere both experience much stronger ozone decreases of up to 5% in the extratropics due to lower NO_*x*_-driven formation and higher NO_*x*_-driven catalytic losses, respectively. These reductions are partially balanced by an ozone increase at 50–100 hPa driven by the updated N + O_2_ reaction, leading to a net decrease of only 0.7% in stratospheric ozone.

In contrast to the decreased ozone, tropospheric HO_*x*_ and H_2_O_2_ burdens all increase slightly (OH +0.3%, HO_2_ + 1.0%, and H_2_O_2_ +1.3%). Increased tropospheric OH is driven largely by the reduced PAA + OH rate coefficient, particularly in the upper troposphere, which is partially offset by the changes to CO + OH and HNO_*z*_ + OH rate coefficients. Higher OH leads to increases in the methane loss rate, causing an overall 0.4% decrease in the methane lifetime against oxidation by tropospheric OH. Higher tropospheric HO_2_ and H_2_O_2_ are driven largely by the accelerated CO + OH rate, with stronger increases in the upper troposphere due to changes in OH + HNO_*z*_ (*z* = 2–4) and over regions with strong biogenic influence due to the revised nitrogen conservation from MVKN. Higher HO_2_ over forests increases the proportion of isoprene-derived peroxy radicals reacting with HO_2_, which correspondingly decreases the fraction that react via the OH-recycling isomerization channel, reducing OH over the Amazon (−1%) and reducing the tropospheric burdens of organic products from the isomerization pathway (e.g., C_5_ hydroperoxyaldehydes, −2.2%).

The cumulative effects of the changes in reactive nitrogen species are shown in Fig. 2, including the absolute change in NO_*x*_ from the base to the updated mechanism (top left) and relative changes for individual species. Maps of absolute changes of the other NO_*y*_ species, which tend to highlight differences in NO_*x*_-rich areas with high anthropogenic influence, are shown in Fig. S11. The net effect of the updates on the tropospheric NO_*x*_ burden is a 1.7% decrease, with a stronger reduction of NO (−2.4%) than NO_2_ (−1.0%). This decrease is dominated by annual average reductions of over 25ppt over China and the Indo-Gangetic Plain, as well as in the tropical upper troposphere, slightly offset by surface NO_*x*_ increases of 1–10ppt in low-NO_*x*_ regions such as the Amazon and high-latitude oceans. Both NO and NO_2_ exhibit strong stratospheric increases (+8% and +7%, respectively), driven in the upper stratosphere by weaker NO_*x*_ loss from a higher N + O_2_ rate coefficient (N originates from NO photolysis, and the N + O_2_ reaction returning NO competes with N + NO producing N_2_) and in the lower stratosphere by changes to the HNO_*z*_ + OH reactions.

Other NO_*y*_ species generally exhibit changes directly attributable to updates in their own sources or sinks. NO_3_, for which loss rate coefficients to reactions with NO_*x*_ and OH are revised downward and the stratospheric source from O + NO_2_ is revised upward, generally increases at the surface (up to +0.5ppt in India and China) and in the stratosphere (+0.4%), although this is offset by a decrease in the midtroposphere. The stratospheric NO_3_ increase is not nearly as strong as that of NO_*x*_, potentially due to faster loss from the increased O + NO_3_ rate coefficient and decreased formation via O_3_ + NO_2_ from the reduction in stratospheric ozone. Changes in N_2_O_5_ follow those of its precursors, NO_2_ and NO_3_, including a decrease of 1.5% in the troposphere and an increase of 6.8% in the stratosphere. Nitrous acid (HNO_2_) is influenced both by changes to its precursors (NO_*x*_) and by a decrease to its loss rate coefficient via reaction with OH; as a result, it decreases in the troposphere (−1.3%), especially over India and China, where decreases to NO_*x*_ are strongest, and increases in the stratosphere (+2.5%). Nitric (HNO_3_) and pernitric (HNO_4_) acids increase slightly in both the troposphere and stratosphere, due to the combined effects of changes in their OH loss rate coefficients and in the mixing ratios of their major precursors.

Among C_1_ species, whose changes are shown in Fig. 3, two effects dominate: the increased CO + OH rate coefficient and the decreased C_1_ stabilized Criegee intermediate (CH_2_OO) + H_2_O rate coefficient. The former causes a 2.7% decrease in the tropospheric CO burden, corresponding to 1–2ppb in the Southern Hemisphere and 2–3ppb in the Northern Hemisphere, with smaller decreases at the continental surface where the change is partially offset by increased CO production from faster oxidation of volatile organics. The latter sharply reduces the formation of hydroxymethyl hydroperoxide (HMHP), the major product of CH_2_OO + H_2_O, decreasing its tropospheric burden by 22%, with the strongest absolute effects (−50 to 75ppt) over tropical and mid-latitude forests, where the ozonolysis of biogenic emissions leads to high CH_2_OO production. Formic acid, a product of the competing reaction of CH_2_OO with the water dimer, increases accordingly by 3.1%. The tropospheric burdens of other C_1_ species increase by 0.1%–1.3% due to indirect effects of faster organic oxidation from the mechanism updates, with the strongest effects at the surface in regions of high biogenic influence and in the tropical upper troposphere.

Figure 4 shows the effects of the mechanism updates on selected larger organic species. Two effects dominate: the reduced PAA + OH rate coefficient and the rebalanced coefficients for primary versus secondary hydrogen abstract from propane by OH. The former leads directly to a 41% increase in the tropospheric PAA burden, with the strongest effects (+150%) in the upper troposphere, where the longer PAA lifetime enables greater transport from lower-tropospheric source regions. Further, reduced regeneration of the peroxyacetyl radical (the product of PAA + OH) is compounded by an increase in its self-reaction rate coefficient; as a result, the products of other peroxyacetyl radical reactions are all decreased, including PAN (−3.8%) and acetic acid (−3.4%). These reductions are enhanced by the updates to PAN cycling rate coefficients and the increased C_2_ Criegee intermediate + SO_2_ coefficient, which decreases acetic acid formation via the competing C_2_ Criegee intermediate + H_2_O reaction. The strongest relative effects of these changes (PAN −12% and acetic acid −6%) are over the tropical oceans, while their strongest absolute effects (−20 and −10ppt, respectively) are over tropical forests.

The adjusted propane oxidation mechanism causes a direct 17% decrease to the tropospheric propane burden due to the overall increased C_3_H_8_ + OH rate coefficient, and this results in a number of knock-on effects from the increased proportion of secondary hydrogen abstraction relative to primary. Tropospheric burdens of the products of the peroxy radical from secondary abstraction all increase (*i*-propyl hydroperoxide +13%, *i*-propyl nitrate +8.6%, and acetone +3.5%), while those from primary abstraction decrease (*n*-propyl hydroperoxide −4.3% and n-propyl nitrate −7.9%). Figure 4 shows the effects on acetone, the most abundant and commonly measured of these products, but the spatial patterns of the other products are similar. While the relative changes in acetone burden (+4%–8%) are evenly spread throughout the troposphere in the Northern Hemisphere, absolute changes are strongest over strong anthropogenic propane source regions (e.g., +200ppt over eastern China). Products of acetone oxidation also exhibit enhancements; for example, the decrease in PAN mixing ratios from the updated PAA + OH coefficient is offset by the increase from higher acetone production, leading to minimal change in the northern extratropics, and the tropospheric methylglyoxal burden increases by 1%. Methylglyoxal is also influenced by the updated temperature dependence of its reaction with OH, leading to higher loss rates (and therefore lower mixing ratios) at colder temperatures, e.g., at high latitudes, and lower loss rates in the warmer tropics.

Finally, Fig. 5 shows the effects of the mechanism updates on selected halogen radical species, which are dominated by changes to the participation of IO in the chlorine and bromine cycles. In general, the decreased IO + ClO rate coefficient leads to higher chlorine reactivity via other pathways and to a general increase in burdens of chlorine radical species; the tropospheric burden of ClO rises by 4.1% and that of Cl_2_O_2_, the major product of ClO + ClO, by 27%, with the strongest effects in the upper troposphere and over the Antarctic. Other chlorine radical species exhibit smaller increases (Cl +1.4% and ClOO +3.0%) with similar spatial patterns, although OClO, a major product of IO + ClO, decreases (−0.7%). Effects in the stratosphere are similar except that ClO and Cl_2_O_2_ decrease, likely due to higher losses from increased NO_*x*_.

In contrast to the effects on chlorine, iodine and bromine radical burdens are generally slightly decreased in the troposphere due to both the updated iodine chemistry with ClO, BrO, or NO_*x*_ and the changes in NO_*x*_ distributions. Tropospheric burdens of most iodine species are moderately lower (I by −1.5%, IO by −0.3%, I_2_O_4_ by −5.8%, INO by −6.0%, and IONO by −9.2%), while those of bromine species are less affected (Br by −0.6%, BrO by −0.7%, BrNO_2_ by −2.9%, and BrNO_3_ by −1.5%), although spatial variability due to adjusted temperature dependence and NO_*x*_ patterns leads to local changes of up to 3% in Br and BrO mixing ratios. In the stratosphere, colder temperatures lead to an increase in the IO + BrO rate coefficient, causing the stratospheric BrO burden to decrease (−1.3%) and those of the IO + BrO products to increase (Br by +1.0% and OIO by +9.7%).

Among the changes not shown in Figs. 1–5, most are minor or are attributable to changes in carbon and nitrogen conservation (Table S2). Despite revisions to the rate coefficients of their reactions with OH, the atmospheric burdens of SO_2_ and OCS barely change (+0.6% and −0.04%, respectively). The addition of a strong temperature dependence to the isoprene ozonolysis rate coefficient also makes little difference: the tropospheric isoprene burden increases by 0.9%, and the decreased fraction of isoprene lost to ozonolysis contributes to the reduced HMHP described above. Most other volatile organic compounds exhibit slight decreases, likely due to the increase in tropospheric OH; for example, the tropospheric burdens of benzene, toluene, xylene, and lumped C_4+_ alkanes decrease by 0.5%–0.6%. The effects on ethylene (−1.8%) and methacrolein (−2.1%) are enhanced by their increased ozonolysis rate coefficients. Carbon-accounting changes cause larger increases to non-specific C_3+_ organic products, e.g., RCHO (C_3+_ aldehydes; +7.4%), ROH (C_3+_ alcohols; +21%), and RP (C_3+_ hydroperoxides; +14%). Finally, the correction of an error in the MVKN yield from its precursor peroxy radical + NO reaction, which had previously caused a net loss of reactive nitrogen from the mechanism, increases the tropospheric burden of MVKN, a species typically underestimated by models (Tsiligiannis et al., 2022), by 39%.

## 5 Conclusions

We updated the chemical mechanism of the GEOS-Chem atmospheric chemistry model, used by a large research community for a wide range of applications, with rate coefficients and product branching ratios from recent kinetic data evaluations. A total of 63 reactions were changed, including 58 on the basis of the 2020 JPL Data Evaluation and 5 on the basis of recent IUPAC recommendations. We further updated 63 reactions to improve carbon and nitrogen conservation between reactants and products. We then quantified the effects of these updates on the GEOS-Chem simulation with reference to version 14.0.1 of the model.

Among the most notable changes to the tropospheric burdens of organic species are a 17% decrease in propane due to updates to its OH chemistry, with accompanying changes to its oxidation products (e.g., a 3.5% increase to acetone); a 41% increase in PAA due to its decreased reaction rate coefficient with OH, with accompanying decreases in its downstream products of PAN (−3.8%) and acetic acid (−3.4%); and a 22% reduction in HMHP due to a reduction in the CH_2_OO + H_2_O rate coefficient, along with a corresponding 3.1% increase in formic acid produced via a competing pathway. A reformulation of the rate coefficient for the CO + OH reaction leads to a 2.7% reduction in the tropospheric CO burden. A total of 11 updates to reactions of reactive nitrogen species result in a 1.7% net decrease in tropospheric NO_*x*_ and a 7% net increase in the stratospheric NO_*x*_ burden, with smaller changes to other NO_*y*_ species. Updates to the rate coefficients of iodine cycling reactions, especially those of IO with ClO, BrO, and NO, cause minor changes to halogen radical distributions, with general small increases in chlorine radical species and decreases in iodine and bromine species. Most secondary effects on trace gases of general wider interest are also minor, including slight reductions in tropospheric burdens of ozone (−0.7%) and increased loadings of formaldehyde (+0.7%) and OH (+0.3%), the latter of which increases the methane loss rate by a corresponding 0.4%. The updated mechanism will more accurately simulate the complex chemical dependencies of key atmospheric species on temperature, pressure, and concentrations of other compounds, and the improved conservation of carbon and nitrogen will facilitate future studies of their overall atmospheric budgets.

## Supplementary Material

as published

## Figures and Tables

**Table 1.** GEOS-Chem reactions updated per JPL and IUPAC recommendations^a^.

Reactants	Products (old mechanism)	Rate coefficient^b^ (old mechanism)	New rate coefficient^b^ and/or products
NO_*y*_ reactions			
OH + NO_3_	HO_2_ + NO_2_	2.2 × 10^−11^	2.0 × 10^−11^
NO_2_ + NO_3_	NO + NO_2_ + O_2_	4.5 × 10^−14^ × *e*^−1260/*T*^	4.35 × 10^−14^ × *e*^−1335/*T*^
O + NO_3_	NO_2_ + O_2_	1.0 × 10^−11^	1.3 × 10^−11^
OH + HNO_2_	NO_2_ + H_2_O	1.8 × 10^−11^ × *e*^−390/*T*^	3.0 × 10^−12^ × *e*^250/*T*^
OH + HNO_4_	NO_2_ + O_2_ + H_2_O	1.3 × 10^−12^ × *e*^380/*T*^	4.5 × 10^−13^ × *e*^610/*T*^
HO_2_ + NO	OH + NO_2_	3.3 × 10^−12^ × *e*^270/*T*^	3.44 × 10^−12^ × *e*^260/*T*^
N + O_2_	NO + O	1.5 × 10^−11^ × *e*^−3600/*T*^	3.3 × 10^−12^ × *e*^−3150/*T*^
NO + NO_3_	2 NO_2_	1.5 × 10^−11^ × *e*^170/*T*^	1.7 × 10^11^ × *e*^125/*T*^
OH + HNO_2_ [+ *M*]	NO_3_ + H_2_O [+ *M*]	*fx* (2.41*e* – 14, 2.69*e* – 17, 6.51*e* – 34)^c^	*f*_a_(3.9*e* – 31, 7.2, 1.5*e* – 13, 4.8, 3.7*e* – 14, −240)^c^
O + NO_2_	NO + O_2_	5.1 × 10^−12^ × *e*^210/*T*^	*f*_b_(3.4*e* – 31, 1.6, 2.3*e* – 11, 0.2, 5.3*e* – 12, −200)^c^
O + NO_2_ [+ *M*]	NO_3_ [+ *M*]	*f*_t_(2.5*e* – 31, 1.8, 2.2*e* – 11, 0.7)^c^	*f*_t_(3.4*e* – 31, 1.6, 2.3*e* – 11, 0.2)^c^
O^1^D-halogen reactions			
O^1^D + HCl	0.67(Cl + OH) + 0.24(ClO + H) + 0.09(HCl + O)	1.5 × 10^−10^	0.66(Cl + OH) + 0.22(ClO + H) + 0.12(HCl + O)
O^1^D + HBr	0.65(Br + OH) + 0.2(HBr + O) + 0.15(BrO + H)	1.5 × 10^−10^	0.6(Br + OH) + 0.2(HBr + O) + 0.2(BrO + H)
O^1^D + CHBr_3_	1.36 Br + 0.68 BrO + 0.32(CHBr_3_ + O)	6.6 × 10^−10^	1.4 Br + 0.7 BrO + 0.3(CHBr_3_ + O)
O^1^D + CCl_4_	2.58 Cl + 0.86 ClO + 0.14(CCl_4_ + O)	3.3 × 10^−10^	2.37 Cl + 0.79 ClO + 0.21(CCl_4_ + O)
O^1^D + CFC11	1.76 Cl + 0.88 ClO + 0.12(CFC11 + O)	2.3 × 10^−10^	1.8 Cl + 0.9 ClO + 0.1(CFC11 + O)
O^1^D + CFC113	1.5 Cl + 0.75 ClO + 0.25(CFC113 + O)	2.32 × 10^−10^	1.8 Cl + 0.9 ClO + 0.1(CFC113 + O)
O^1^D + CFC114	0.75(Cl + ClO) + 0.25(CFC114 + O)	1.3 × 10^−10^ × *e*^−25/*T*^	1.3 × 10^−10^ × *e*^25/*T*^; 0.95 Cl + 0.85ClO + 0.1(CFC114 + O)
O^1^D + CFC115	0.7(CFC115 + O) + 0.3 ClO	5.4 × 10^−11^ × *e*^−30/*T*^	5.4 × 10^−11^ × *e*^30/*T*^; 0.86 ClO + 0.14(CFC115 + O)
O^1^D + HCFC22	0.55 ClO + 0.28(HCFC22 + O) + 0.17 Cl	1.02 × 10^−10^	0.56 ClO + 0.25(HCFC22 + O) + 0.19 Cl + 0.05 OH
O^1^D + HCFC142b	0.74 ClO + 0.26(HCFC142b + O)	2.0 × 10^−10^	0.65 ClO + 0.35(HCFC142b + O)
O^1^D + H1211	0.36(H1211 + O) + 0.33(ClO + Br) + 0.31(Cl + BrO)	1.5 × 10^−10^	0.35(H1211 + O) + 0.34(ClO + Br) + 0.31(Cl + BrO)
O^1^D + H1301	0.59(H1301 + O) + 0.41 BrO	1.0 × 10^−10^	0.55(H1301 + O) + 0.45 BrO
O^1^D + CH_3_Cl	n/a	n/a	2.6 × 10^−10^; 0.9 CH_3_OO + 0.46 ClO + 0.35 Cl + 0.1(0 + CH_3_Cl) + 0.09 H
Ozonolysis reactions			
Ethene + O_3_	CH_2_O + CH_2_OO	9.1 × 10^−15^ × *e*^−2580/*T*^	1.2 × 10^−14^ × *e*^−2630/*T*^
Propene + O_3_	0.5 CH_3_CHO + 0.5 CH_2_O + …	5.5 × 10^−15^ × *e*^−1880/*T*^	6.5 × 10^−15^ × *e*^−1900/*T*^
Methacrolein + O_3_	0.88 CH_3_C(O)CHO + …	1.4 × 10^−15^ × *e*^−2100/*T*^	1.5 × 10^−15^ × *e*^−2110/*T*^
Isoprene + O_3_	0.827 CH_2_O + 0.58 CH_2_OO + …	1.3 × 10^−17^	1.1 × 10^−14^ × *e*^−2000/*T*^
OH + organic reactions			
propane + OH	CH_3_CH(OO)CH_3_	*f*_P_(5.87, 0.64, −816)^c^	8.54 × 10^−13^ × *e*^−19/^^*T*^ × ($\frac{298}{T}$)^1.54^
propane + OH	CH_3_CH_2_CH_2_OO	*f*_p_(0.17, −0.64, 816)^c^	1.97 × 10^−12^ × *e*^−675/^^*T*^ × ($\frac{298}{T}$)^1.23^
C_3+_ alcohol + OH	C_3+_ aldehyde + HO_2_	4.6 × 10^−12^ × *e*^70/*T*^	4.4 × 10^−12^ × *e*^70/*T*^
CH_2_Cl_2_ + OH	2 Cl + HO_2_	2.61 × 10^−12^ × *e*^−944/*T*^	1.92 × 10^−12^ × *e*^−880/*T*^
CHCl_3_ + OH	3 Cl + HO_2_	4.69 × 10^−12^ × *e*^−1134/*T*^	2.2 × 10^−12^ × *e*^−920/*T*^
CH_3_C(O)CHO + OH	CH_3_CO_3_ + CO	1.5 × 10^−11^	1.9 × 10^−12^ × *e*^575/^^*T*d^
CH_3_C(O)CH_2_CH_3_ + OH	CH_3_C(O)CH_2_CH_2_OO	1.3 × 10^−12^ × *e*^−25/*T*^	1.5 × 10^−12^ × *e*^−90/^^*T*d^
CH_3_CO_3_H + OH	CH_3_CO_3_	6.13 × 10^−13^ × *e*^200/*T*^	3.0 × 10^−14^; 0.78 CH_3_CO_3_^d^ + 0.22(OH + CO_2_ + CH_2_O)
CH_3_C(O)CH_2_OH + OH	CH_3_C(O)CHO + HO_2_	*f*_HA_(2.15*e* – 12, 305)^c^	*f*_HA_(2.0*e* – 12, 320)^c^
CH_3_C(O)CH_2_OH + OH	0.5(CH_3_CO_2_H + HCOOH) + …	*f*_HB_(2.15*e* – 12, 305)^c,d^	*f*_HB_(2.0*e* – 12, 320)^c,d^
Cl + organic reactions			
Cl + CH_2_O	CO + HCl + HO_2_	7.32 × 10^−11^ × *e*^−30/*T*^	8.1 × 10^−11^ × *e*^−30/^^*T*^
Cl + Acetone	HCl + CH_3_C(O)CH_2_OO	7.7 × 10^−11^ × *e*^−1000/^^*T*^	1.63 × 10^−11^ × *e*^−610/^^*T*^
Cl + CH_3_Cl	CO + 2 HCl + HO_2_	2.17 × 10^−11^ × *e*^−1130/^^*T*^	2.03 × 10^−11^ × *e*^−1110/^^*T*^
Cl + CH_2_Cl_2_	CO + HCl + HO_2_ + 2 Cl	1.24 × 10^−12^ × *e*^−1070/^^*T*^	7.4 × 10^−12^ × *e*^−910/^^*T*^
Cl + CHCl_3_	CO + HCl + HO_2_ + 3 Cl	3.77 × 10^−12^ × *e*^−1011/^^*T*^	3.3 × 10^−12^ × *e*^−990/^^*T*^
Peroxy radical (RO_2_) reactions			
CH_3_CO_3_ + CH_3_CO_3_	2 CH_3_OO	2.5 × 10^−12^ × *e*^500/^^*T*^	2.9 × 10^−12^ × *e*^500/*T*^
CH_3_C(O)CH_2_OO + NO	CH_3_CO_3_ + CH_2_O + NO_2_	2.8 × 10^−12^ × *e*^300/^^*T*^	2.9 × 10^−12^ × *e*^300/^^*T*^
CH_3_CH_2_OO + HO_2_	CH_3_CH_2_OOH	7.4 × 10^−13^ × *e*^700/^^*T*^	7.5 × 10^−13^ × *e*^700/^^*T*^
OTHRO2^e^ + HO_2_	CH_3_CH_2_OOH	7.4 × 10^−13^ × *e*^700/^^*T*^	7.5 × 10^−13^ × *e*^700/^^*T*^
CH_3_CO_3_ + NO_2_ [+ *M*]	PAN [+ *M*]	*f*_t_(9.7*e* – 29, 5.6, 9.3*e* – 12, 1.5)^c^	*f*_t_(7.3*e* – 29, 4.1, 9.5*e* – 12, 1.6)^c^
Criegee intermediate reactions			
CH_2_OO + NO_2_	CH_2_O + NO_3_	1.0 × 10^−15^	4.25 × 10^−12^
CH_2_OO + SO_2_	CH_2_O + SO_4_	3.7 × 10^−11^	3.8 × 10^−11^
CH_2_OO + H_2_O	0.73 HMHP + 0.21 HCOOH + …	1.7 × 10^−15^	2.8 × 10^−16^
CH_3_CH_2_OO + SO_2_	CH_3_CHO + SO_4_	7.0 × 10^−14^	2.65 × 10^−11^
Iodine reactions			
IO + NO	I + NO_2_	9.1 × 10^−12^ × *e*^240/^^*T*^	8.6 × 10^−12^ × *e*^230/^^*T*^
IO + ClO	0.801 I + 0.56 OClO + …	8.93 × 10^−12^ × *e*^280/*T*^	4.82 × 10^−12^ × *e*^280/*T*^
IO + BrO	Br + 0.8 OIO + …	1.5 × 10^−11^ × *e*^510/^^*T*^	5.5 × 10^−12^ × *e*^760/^^*T*^
I + O_3_	IO + O_2_	2.3 × 10^−11^ × *e*^−870/^^*T*^	2.0 × 10^−11^ × *e*^−830/^^*T*^
I + NO [+ *M*]	INO [+ *M*]	*f*_t_(1.8*e* – 32, 1, 1.77*e* – 11, 0)^c^	*f*_t_(1.8*e* – 32, 1, 1.7*e* – 11, 0)^c^
I + NO_2_ [+ *M*]	IONO [+ *M*]	*f*_t_(3*e* – 31, 1, 6.6*e* – 11, 0, 0.63)^c^	*f*_t_(3*e* – 31, 1, 6.6*e* – 11, 0, 0.6)^c^
Other reactions			
OCS + OH	CO_2_ + SO_2_	1.1 × 10^−13^ × *e*^−1200/^^*T*^	7.2 × 10^−14^ × *e*^−1070/^^*T*^
CO + OH [+ *M*]	HO_2_ + CO_2_ [+ *M*]	*f*_c_(5.9*e* – 33, 1.1*e* – 12, 1.5*e* – 13, 2.1*e*9)^c^	*f*_a_(6.9*e* – 33, 2.1, 1.1*e* – 12, −1.3, 1.85*e* – 13, 65)^c^
SO_2_ + OH [+ *M*]	SO_4_ + HO_2_ [+ *M*]	*f*_t_(3.3*e* – 31, 4.3, 1.6*e* – 12, 0)^c^	*f*_t_(2.9*e* – 31, 4.1, 1.7*e* – 12, −0.2)^c^
H + O_2_ [+ *M*]	HO_2_ [+ *M*]	*f*_t_(4.4*e* – 32, 1.3, 7.5*e* – 11, −0.2)^c^	*f*_t_(5.3*e* – 32, 1.8, 9.5*e* – 11, −0.4)^c^
PAN [+ *M*]	CH_3_CO_3_ + NO_2_ [+ *M*]	*f*_d_(9.7*e* – 29, 5.6, 9.3*e* – 12, 1.5)^c^	*f*_d_(7.3*e* – 29, 4.1, 9.5*e* – 12, 1.6)^c^

a Updates are from JPL Data Evaluation recommendations unless denoted otherwise by a table footnote in Column 4; GEOS-Chem names for chemical species listed here by formula or common name are given in Table S1.

b In units cm^3^ molecule^−1^ s^−1^ unless otherwise noted; *T* is temperature in K.

c Formulas for the rate coefficients of propane + OH (*f*_P_), hydroxyacetone + OH (*f*_HA_ & *f*_HB_), termolecular reactions (*f*_t_), dissociations (*f*_d_, units s^−1^), activation reactions (*f*_a_ & *f*_b_), and previous GEOS-Chem parameterizations of OH + HNO_3_ (*f*_*x*_) and OH + CO (*f*_c_) are given in Sect. S1 in the Supplement.

d Per IUPAC recommendations.

e OTHRO2 represents functionalized C_2_ peroxy radicals aside from CH_3_CH_2_OO. n/a – not applicable

## Data Availability

KPP (Lin et al., 2023; available at https://kpp.readthedocs.io/, last access: 24 April 2023) and GEOS-Chem (https://geoschem.github.io/, last access: 24 April 2023; DOI: https://doi.org/10.5281/zenodo.7271974, The International GEOS-Chem User Community, 2022) are both available online for public use. GEOS-Chem mechanism inputs and simulation outputs for this work are available online at https://doi.org/10.7910/DVN/IDYV3E (Bates, 2023).
