# Supplementary material for: Impacts of updated reaction kinetics on the global GEOS-Chem simulation of atmospheric chemistry
Source: Geosci Model Dev. Author manuscript; Available in PMC 2024 Mar 20. (PMC10953788; doi:10.5194/gmd-17-1511-2024)
Supplement: as published [file NIHMS1969923-supplement-as_published.pdf]

Supplement of Geosci. Model Dev., 17, 1511–1524, 2024
https://doi.org/10.5194/gmd-17-1511-2024-supplement
© Author(s) 2024. CC BY 4.0 License.

Geoscientific
Model Development
Open Access

Supplement of

Impacts of updated reaction kinetics on the global GEOS-Chem simulation of atmospheric chemistry

Kelvin H. Bates et al.

Correspondence to: Kelvin H. Bates (kelvin.bates@noaa.gov)

The copyright of individual parts of the supplement might differ from the article licence.

Contents

S1 Rate coefficient formulas **S1**

S2 GEOS-Chem species names and abbreviations **S3**

S3 Figures of updated rate coefficients **S5**

S4 GEOS-Chem updates not included in Table 1 **S13**

S5 Additional impacts of updates **S20**

S1 Rate coefficient formulas

This section provides formulas for calculating the more complex rate coefficients used in Table 1 in the main text. In each of the following equations, T represents temperature in Kelvin, M represents the number density of air in molecules cm^{-3}, and the rate coefficients returned by the individual formulas are in units of cm^3 molecule^{-1} s^{-1} unless otherwise noted.

The formula for the rate coefficient of propane + OH, f_P, is given by the following equation:

$$f_P(a,b,c) = 7.6 \times 10^{-12} \times e^{(-585/T)} / (1 + a \times (\frac{300}{T})^b \times e^{(c/T)}) \quad \text{(S1)}$$

The formulas for the rate coefficients of the two hydroxyacetone + OH branches, f_{HA} and f_{HB}, are given by the following equations, where MAX[x,y] returns the maximum of the values of x or y:

$$f_{HA}(a,b) = a \times e^{(b/T)} \times \mathrm{MAX}[0,(1 - 23.7 \times e^{(-T/60)})] \quad \text{(S2)}$$

$$f_{HB}(a,b) = a \times e^{(b/T)} \times (1 - \mathrm{MAX}[0,(1 - 23.7 \times e^{(-T/60)})]) \quad \text{(S3)}$$

A number of termolecular association reactions appear in Table 1, with rate coefficients given by the following equation. This version includes a multiplication by M in order to return a value with units cm^3 molecule^{-1} s^{-1} that can be treated like the rate coefficient of a standard bimolecular reaction. When the formula does not appear with a fifth argument (z), the value

can be assumed as 0.6.

$$f_t(a,b,x,y,z) = \frac{M \times a \times (\frac{298}{T})^b}{1 + \frac{M \times a \times (\frac{298}{T})^b}{x \times (\frac{298}{T})^y}} \times z^{[1/(1+[\log_{10}(\frac{M \times a \times (298/T)^b}{x \times (298/T)^y})]^2)]} \tag{S4}$$

Some termolecular association reactions also have an activation pathway, whereby the excited intermediate can fall apart instead of yielding the recombination production. While these appear to be simple bimolecular reactions, their coupling with the association branch produces a pressure dependence. Similarly to the termolecular association reactions, the rate coefficient formula used here includes a multiplication by M in order to return a value with units cm^3 molecule^{-1} s^{-1} that can be treated like a standard bimolecular reaction rate coefficient. The activation pathway rate coefficient is given by the following equation:

$$f_b(a,b,x,y,c,d) = c \times e^{(-d/T)} \times (1 - \frac{f_t(a,b,x,y)}{x \times (\frac{298}{T})^y}) \tag{S5}$$

For the reactions of OH + CO and OH + HNO_3, both the association and activation pathways yield identical products, so their effective rate coefficient can be calculated as the sum of the two pathways:

$$f_a(a,b,x,y,c,d) = f_b(a,b,x,y,c,d) + f_t(a,b,x,y) \tag{S6}$$

The PAN dissociation rate coefficient (f_d, units s^{-1}) is calculated by dividing the equilibrium constant by the forward termolecular association rate coefficient, as in the following equation:

$$f_d(a,b,x,y) = \frac{f_t(a,b,x,y,0.6)}{9.3 \times 10^{-29} \times e^{(14000/T)}} \tag{S7}$$

Finally, prior to these updates, GEOS-Chem used unique rate coefficient formulas for the reactions of OH + HNO_3 and OH + CO, given by the following formulas:

$$f_x(a,b,c) = a \times e^{(460/T)} + M \times c \times e^{(1335/T)} / (1 + \frac{M \times c \times e^{(1335/T)}}{b \times e^{(2199/T)}}) \tag{S8}$$

$$f_c(a,b,c,d) = f_t(a,1.0,b,-1.3) + f_t(c,0,d,-6.1)/M \tag{S9}$$

S2 GEOS-Chem species names and abbreviations

Table S1 provides common names and formulas for the species whose GEOS-Chem abbreviations are used in descriptions of updated reactions (Table 1, Tables S2-S4). For a complete list of GEOS-Chem species, including those listed in Table S5, see the GEOS-Chem wiki.

Table S1: Species names and abbreviations from GEOS-Chem used in this work[a]

GEOS-Chem name	Formula	Other names or description
A3O2	$CH_3CH_2CH_2OO$	Primary peroxy radical from propane
ACET	$CH_3C(O)CH_3$	Acetone
ACTA	CH_3CO_2H	Acetic acid
ALD2	CH_3CHO	Acetaldehyde
AROMP4	$C_4H_4O_3$[b]	Generic C_4 product from aromatic oxidation
AROMP5	$C_5H_7O_3$[b]	Generic C_5 product from aromatic oxidation
AROMRO2	N/A	Generic peroxy radical from aromatic oxidation
ATO2	$CH_3C(O)CH_2OO$	Peroxy radical from acetone
ATOOH	$CH_3C(O)CH_2OOH$	Hydroperoxy-acetone
BENZ	C_6H_6	Benzene
B3O2	$CH_3CH(OO)CH_3$	Secondary peroxy radical from propane
EOH	CH_3CH_2OH	Ethanol
ETO2	CH_3CH_2OO	Ethylperoxy radical
GLYC	$HOCH_2CHO$	Glycolaldehyde
GLYX	$C_2H_2O_2$	Glyoxal
HAC	$CH_3C(O)CH_2OH$	Hydroxyacetone
HMHP	$HOOCH_2OH$	Hydroxymethyl hydroperoxide
HMML	$C_4H_6O_3$	Hydroxymethyl-methyl-α-lactone
HONIT	$C_{10}H_{17}NO_4$	Second-generation monoterpene organonitrates
HPALD2	$HOOCH_2C(CH_3)=CHCHO$	1,4-C_5-hydroperoxyaldehyde
ICN	$C_5H_7NO_4$	Isoprene carbonyl nitrates
ICNOO	$C_5H_8NO_7$	Peroxy radicals from isoprene carbonyl nitrates
ICPDH	$C_5H_{10}O_5$	Isoprene dihydroxy hydroperoxycarbonyl
IDN	$C_5H_8N_2O_6$	Lumped isoprene dinitrates
IHN1	$C_5H_9NO_4$	Isoprene 4,1-hydroxynitrate
INA	$C_5H_8NO_4$	Alkoxy radical from INO2D
INO2B	$C_5H_8NO_5$	β-peroxy radicals from isoprene + NO_3
INO2D	$C_5H_8NO_5$	δ-peroxy radicals from isoprene + NO_3
IONITA	$C_5H_9NO_4$[b]	Aerosol-phase organonitrates from isoprene
ITCN	$C_5H_9NO_7$	Lumped tetrafunctional isoprene carbonyl-nitrates
ITHN	$C_5H_{11}NO_7$	Lumped tetrafunctional isoprene hydroxynitrates
KO2	$C_4H_5O_3$[b]	Peroxy radicals from C_{3+} ketones
LIMO	$C_{10}H_{16}$	Limonene
LIMO2	$C_{10}H_{17}O_3$	Peroxy radical from limonene + OH
MACR	$CH_2=C(CH_3)CHO$	Methacrolein

Continued on next page

Continued from previous page

GEOS-Chem name	Formula	Other names or description
MACR1OO	CH_2=C(CH_3)C(O)OO	Peroxyacyl radical from methacrolein + OH
MACR1OOH	CH_2=C(CH_3)C(O)OOH	Peracid from MACR1OO
MAP	$CH_3C(O)O_2H$	Peroxyacetic acid
MCO3	$CH_3C(O)O_2$	Peroxyacetyl radical
MCRDH	$C_4H_8O_3$	Dihydroxy-methacrolein
MCRENOL	$C_4H_6O_2$	Lumped enols from MVK/MACR oxidation
MCRHN	$HOCH_2C(ONO_2)(CH_3)CHO$	Hydroxynitrate from MACR
MCRHNB	$O_2NOCH_2C(OH)(CH_3)CHO$	Hydroxynitrate from MACR
MEK	$CH_3C(O)CH_2CH_3$	Methyl ethyl ketone
MGLY	$CH_3C(O)CHO$	Methylglyoxal
MONITS	$C_{10}H_{17}NO_4$	Saturated first-generation monoterpene organonitrates
MONITU	$C_{10}H_{17}NO_4$	Unsaturated first-generation monoterpene organonitrates
MTPA	$C_{10}H_{16}$	Lumped α-pinene, β-pinene, sabinene, and carene
MTPO	$C_{10}H_{16}$	Lumped terpenes aside from MTPA + LIMO
MVK	CH_2=CHC(O)CH_3	Methyl vinyl ketone
MVKHCB	$C_4H_6O_3$	Hydroxy-carbonyl from MVK
MVKN	$C_4H_7NO_5$	Hydroxynitrate from MVK
MVKOHOO	$C_4H_7O_4$	Peroxy radical from MVK + OH
MO2	CH_3OO	Methylperoxy radical
MP	CH_3OOH	Methyl hydroperoxide
NPHEN	$C_6H_5NO_3$	Nitrophenols
OLND	$C_{10}H_{16}NO_5$	Peroxy radical from monoterpenes + NO_3
OLNN	$C_{10}H_{16}NO_5$	Peroxy radical from monoterpenes + NO_3
OTHRO2	$CH_3CH_2OO^b$	C_2 Peroxy radicals from sources besides ethane
PAN	$CH_3C(O)OONO_2$	Peroxyacetyl nitrate
PHEN	C_6H_6O	Phenol
PIO2	$C_{10}H_{17}O_3$	Peroxy radical from monoterpenes
PIP	$C_{10}H_{18}O_3$	Hydroperoxide from PIO2
PO2	$HOCH_2CH(OO)CH_3$	Peroxy radical from propene + OH
PRN1	$O_2NOCH_2CH(OO)CH_3$	Peroxy radical from propene + NO_3
PROPNN	$CH_3C(O)CH_2ONO_2$	Propanone nitrate
PYAC	$C_3H_4O_3$	Pyruvic acid
R4N1	$C_4.5H_8NO_5{}^b$	Peroxy radical from C_{4+} alkyl nitrates
R4O2	$C_4.5H_9O_2{}^b$	Peroxy radicals from C_{4+} alkanes
R4P	$C_4.5H_9OOH^b$	C_{4+} hydroperoxides
RCHO	$CH_3CH_2CHO^b$	C_{3+} aldehydes
RCO3	$CH_3CH_2C(O)O_2$	Peroxypropionyl radical
RCOOH	$C_2H_5C(O)OH^b$	C_{3+} acids
ROH	$C_3H_7OH^b$	C_{3+} alcohols
RP	$CH_3CH_2C(O)O_2H$	Peracid from RCO3
SOAGX	$C_2H_2O_2$	Aerosol-phase glyoxal
SOAIE	$C_5H_{10}O_3$	Aerosol-phase isoprene epoxydiols (IEPOX)

[a] Species with GEOS-Chem names equivalent to their formulas are not included here.

[b] Effective formula (for C/N balance) for species that represent a lumped group of compounds.

S3 Figures of updated rate coefficients

Figures S1-S9 show the absolute and relative changes made to GEOS-Chem reaction rate coefficients in this work. Reactions are grouped and organized as in Table 1 of the main text. For most reactions, values are shown across the temperature range 200 – 300 K; for pressure-dependent reactions, rate coefficients are instead shown as a function of altitude across the range 0 – 10 km, where the dependence of both temperature and pressure on altitude are shown in Figure S10. Reactions of the Criegee radicals are excluded due to their lack of temperature dependence.

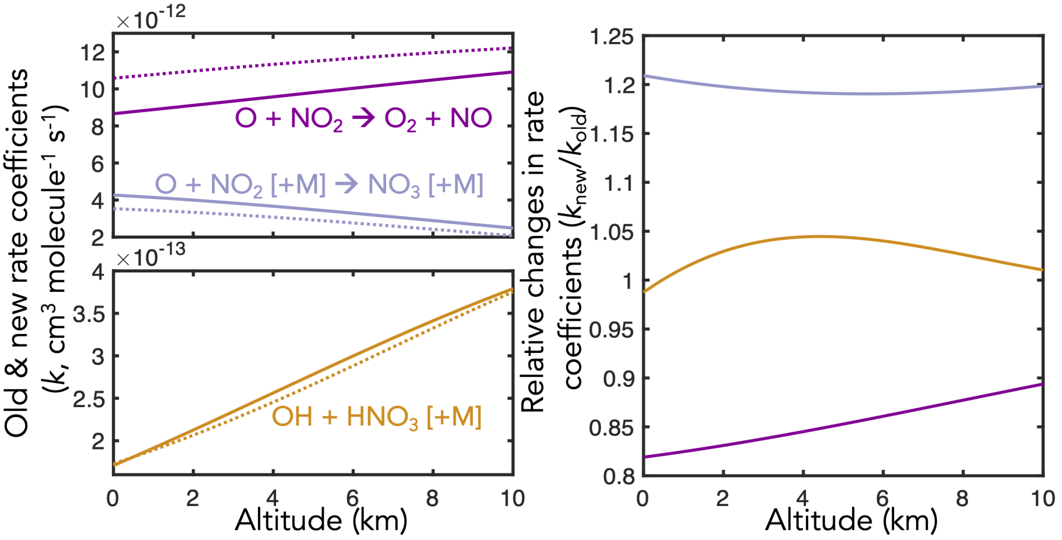

Figure S1. Base and updated rate coefficients (left) and relative difference (right) for pressure- and temperature-dependent reactions in the NO_y family as functions of altitude (where variation of temperature and pressure with altitude is shown in Figure S10). Reactions are separated onto different scales in the top and bottom panels to better show their ranges.

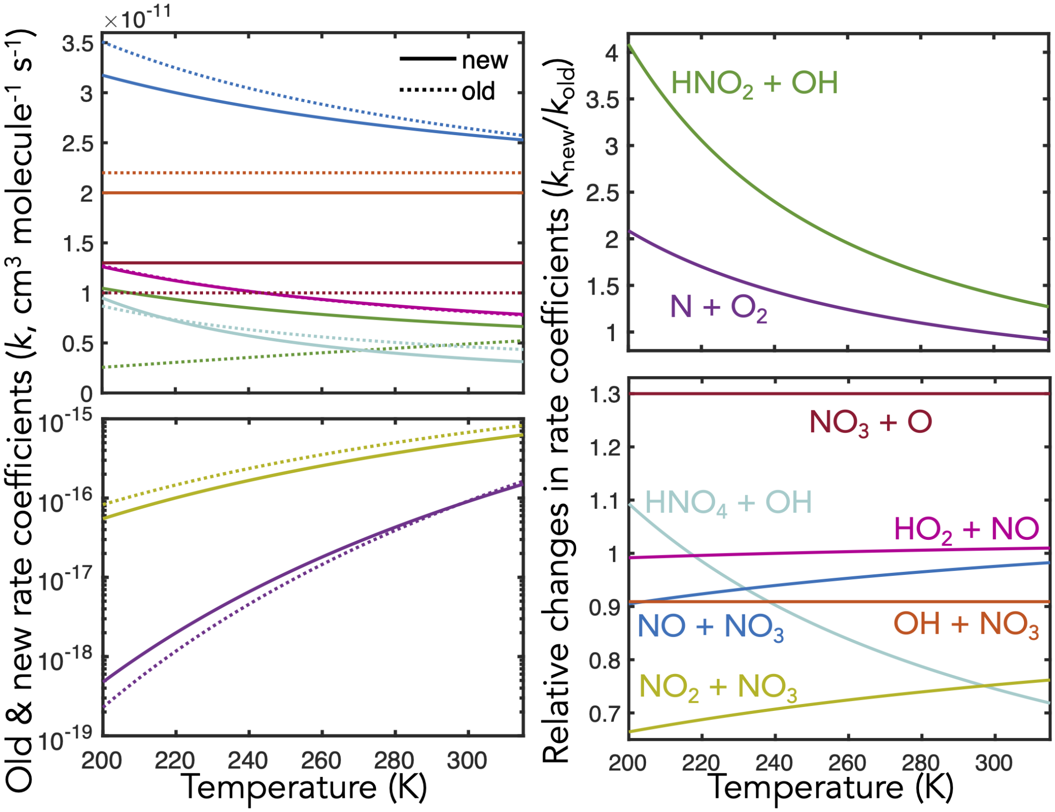

Figure S2. Base and updated rate coefficients (left) and relative difference (right) for temperature-dependent reactions in the NO_y family as functions of temperature. Reactions are separated onto different scales in the top and bottom panels to better show their ranges.

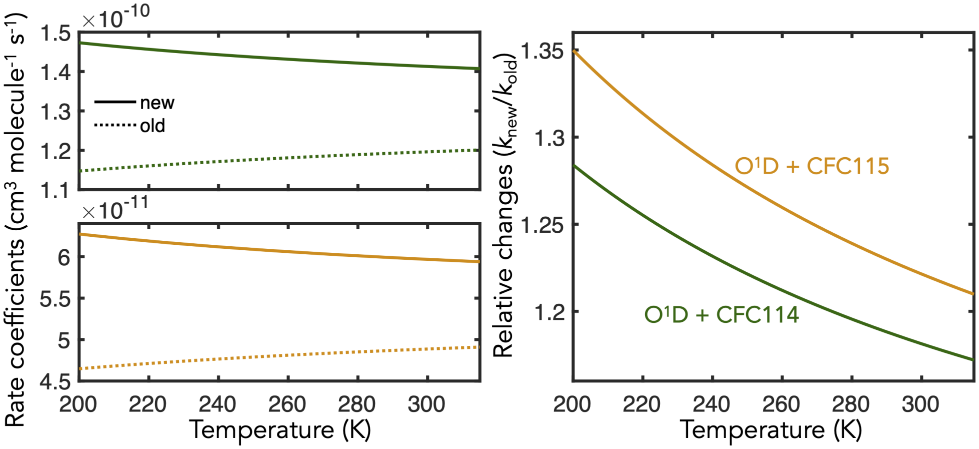

Figure S3. Base and updated rate coefficients (left) and relative difference (right) for reactions of O^1D as functions of temperature. Reactions are separated onto different scales in the top and bottom panels to better show their ranges.

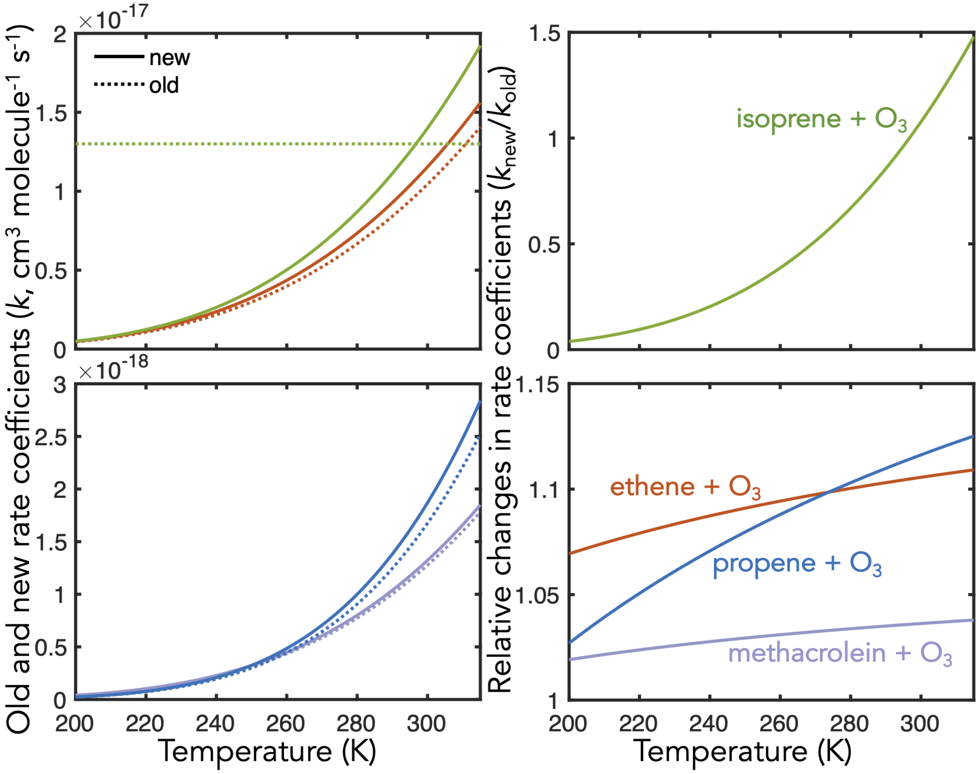

Figure S4. Base and updated rate coefficients (left) and relative difference (right) for organic ozonolysis reactions as functions of temperature. Reactions are separated onto different scales in the top and bottom panels to better show their ranges.

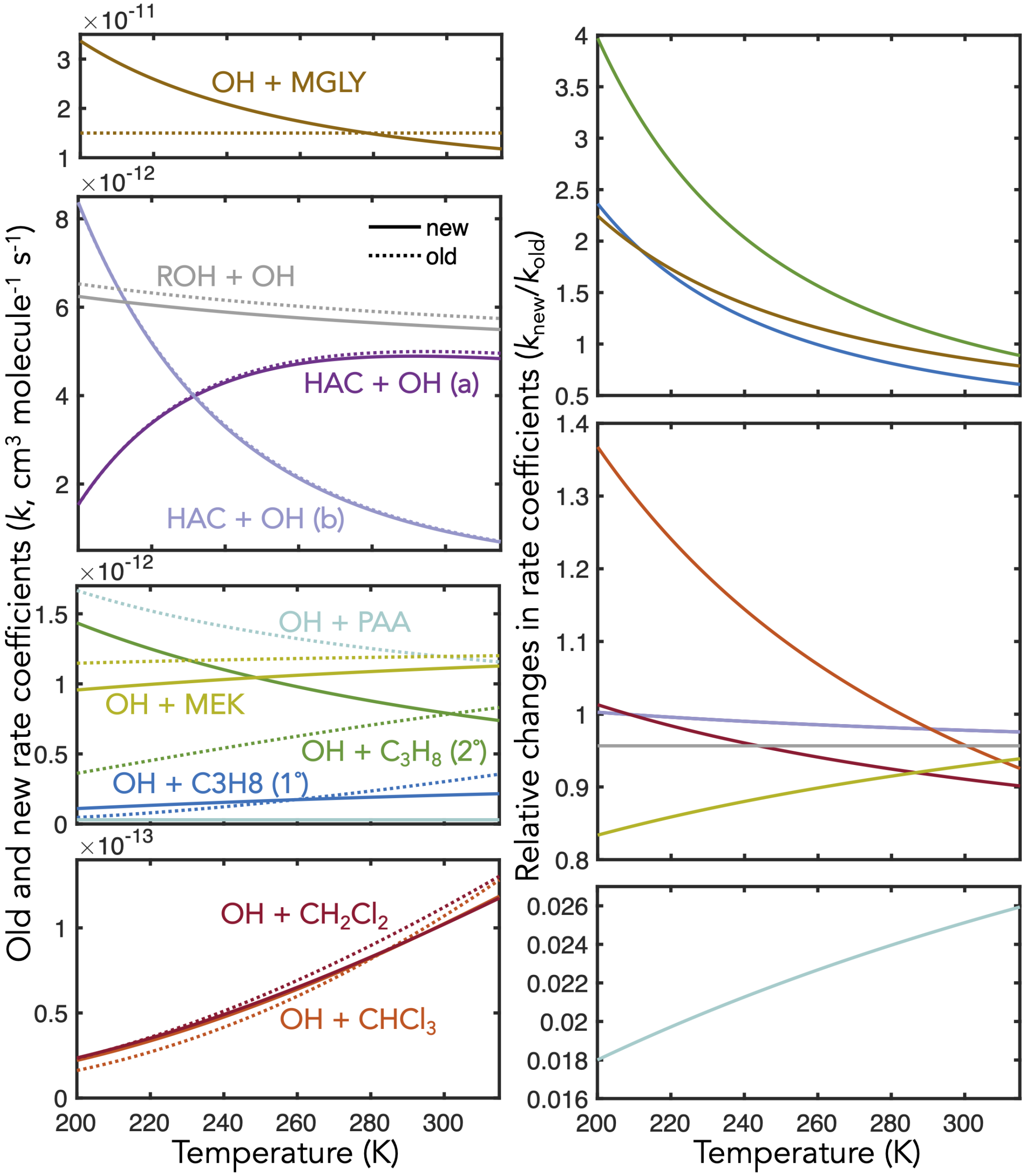

Figure S5. Base and updated rate coefficients (left) and relative difference (right) for reactions of OH with organics as functions of temperature. Reactions are separated onto different scales across vertical panels to better show their ranges. MGLY = methylglyoxal, ROH = C_{3+} alcohols, HAC = hydroxyacetone (parenthetical a and b refer to the two abstraction pathways, where a leads to methylglyoxal and b to fragmentation; note that their relative differences overlap), PAA = peroxyacetic acid, MEK = methyl ethyl ketone, and the parenthetical 1 and 2 after C_3H_8 refer to the primary and secondary abstraction pathways.

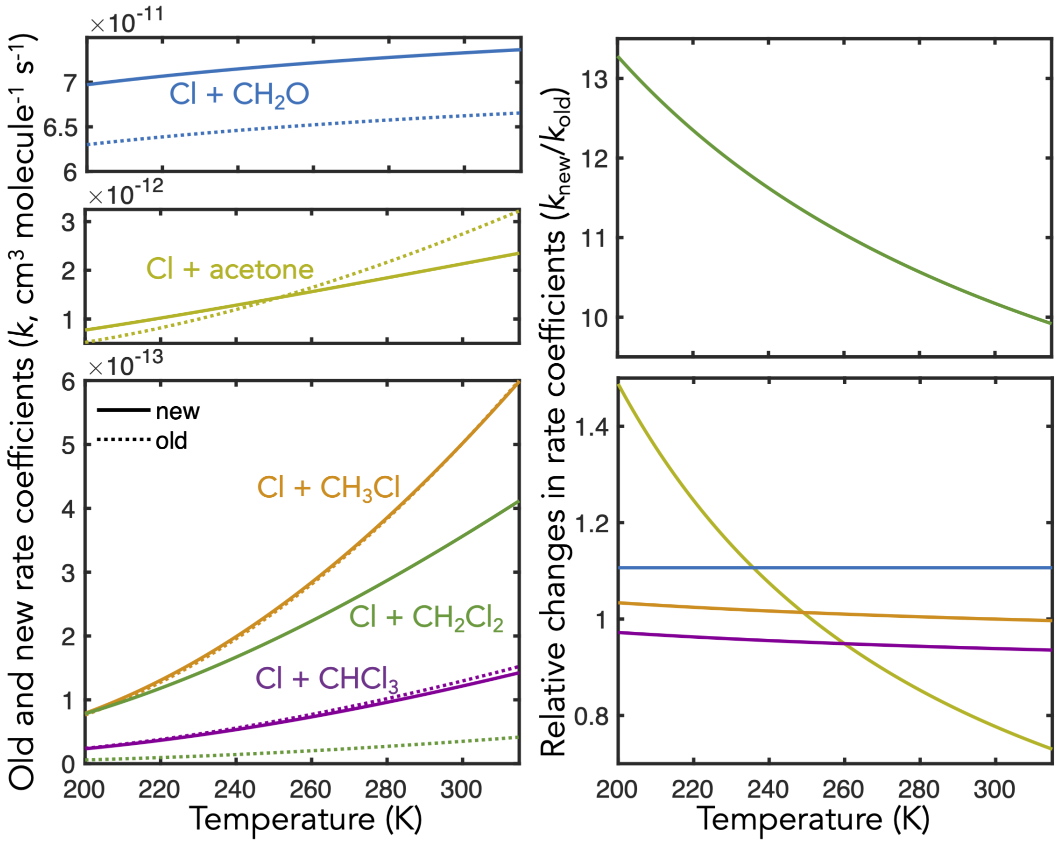

Figure S6. Base and updated rate coefficients (left) and relative difference (right) for reactions of Cl radicals with organics as functions of temperature. Reactions are separated onto different scales across vertical panels to better show their ranges.

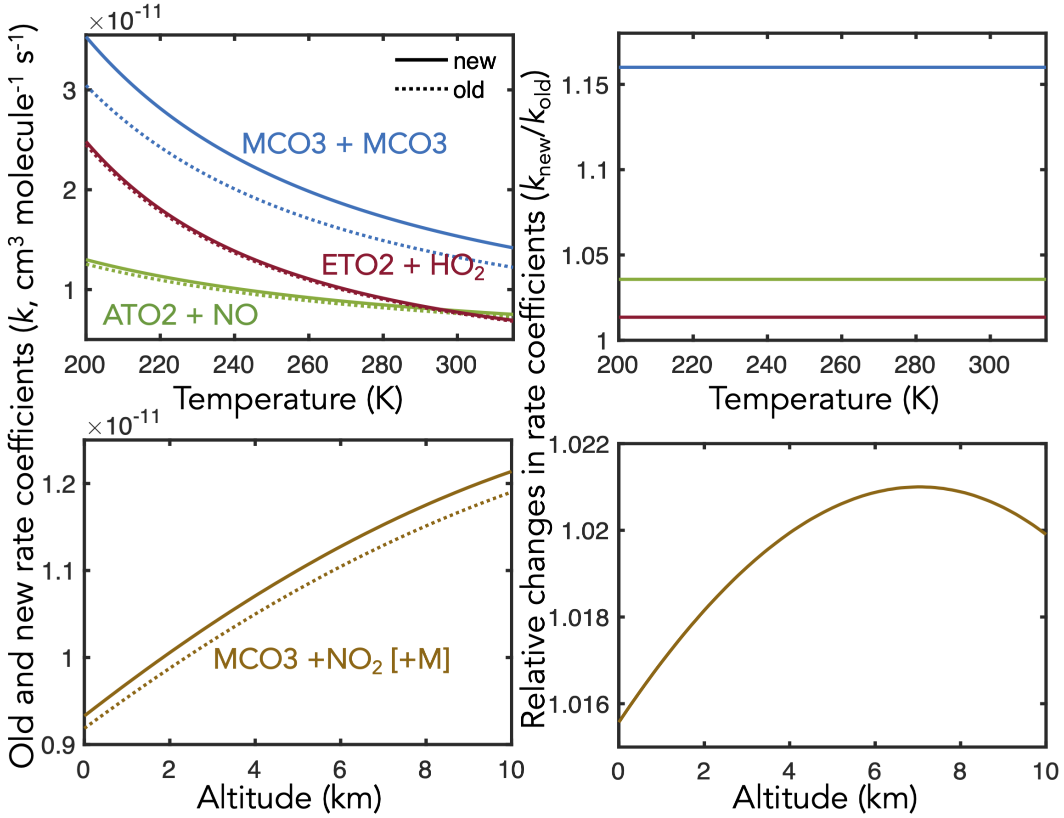

Figure S7. Base and updated rate coefficients (left) and relative difference (right) for reactions of peroxy radicals as functions of temperature (top) and, for termolecular reactions, altitude (bottom, where variation of temperature and pressure with altitude is shown in Figure S10). MCO3 = peroxyacetyl radical, ETO2 = ethylperoxy radical, ATO2 = peroxy radical from acetone + OH.

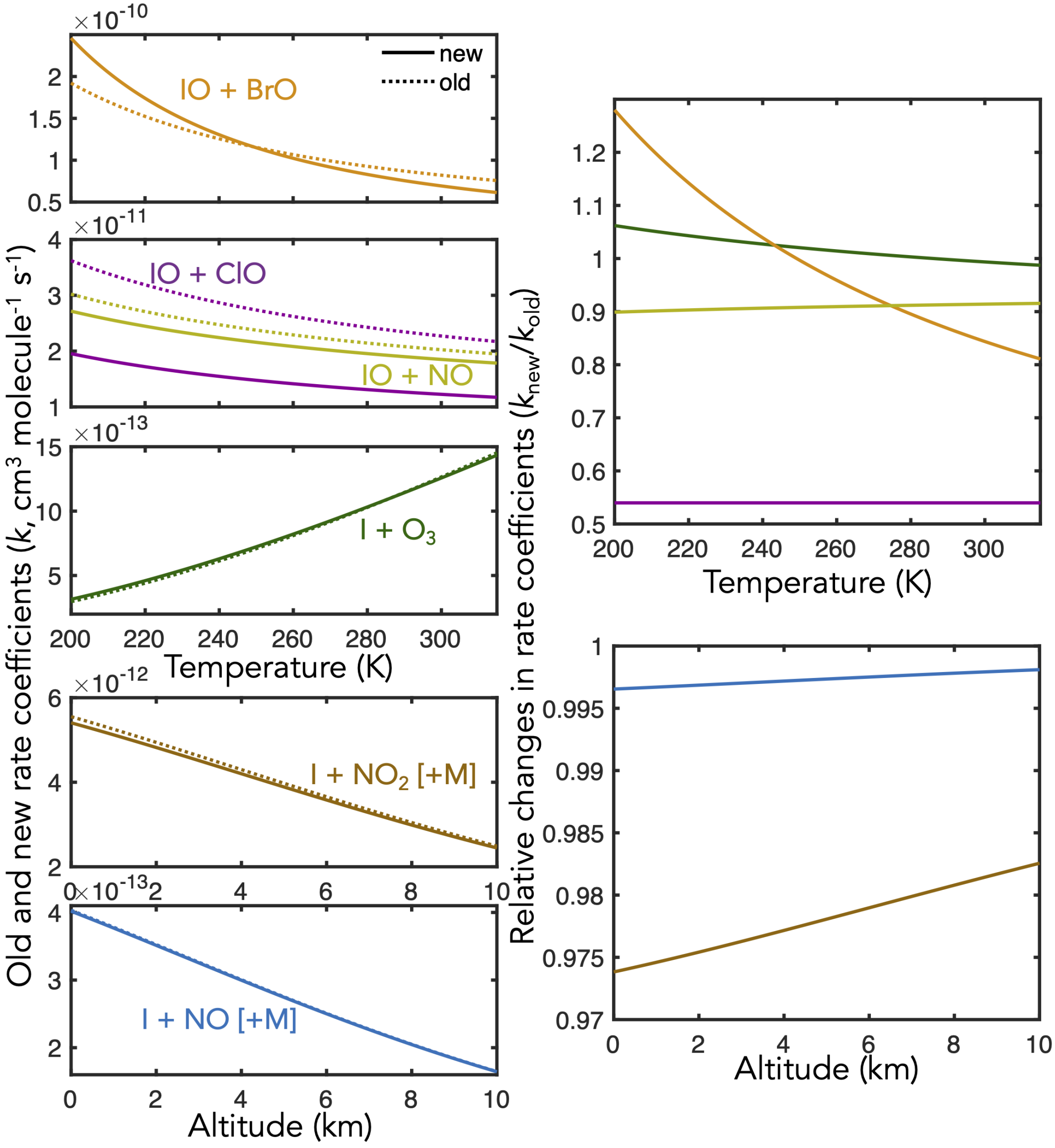

Figure S8. Base and updated rate coefficients (left) and relative difference (right) for reactions of iodine species as functions of temperature (top) and, for termolecular reactions, altitude (bottom, where variation of temperature and pressure with altitude is shown in Figure S10).

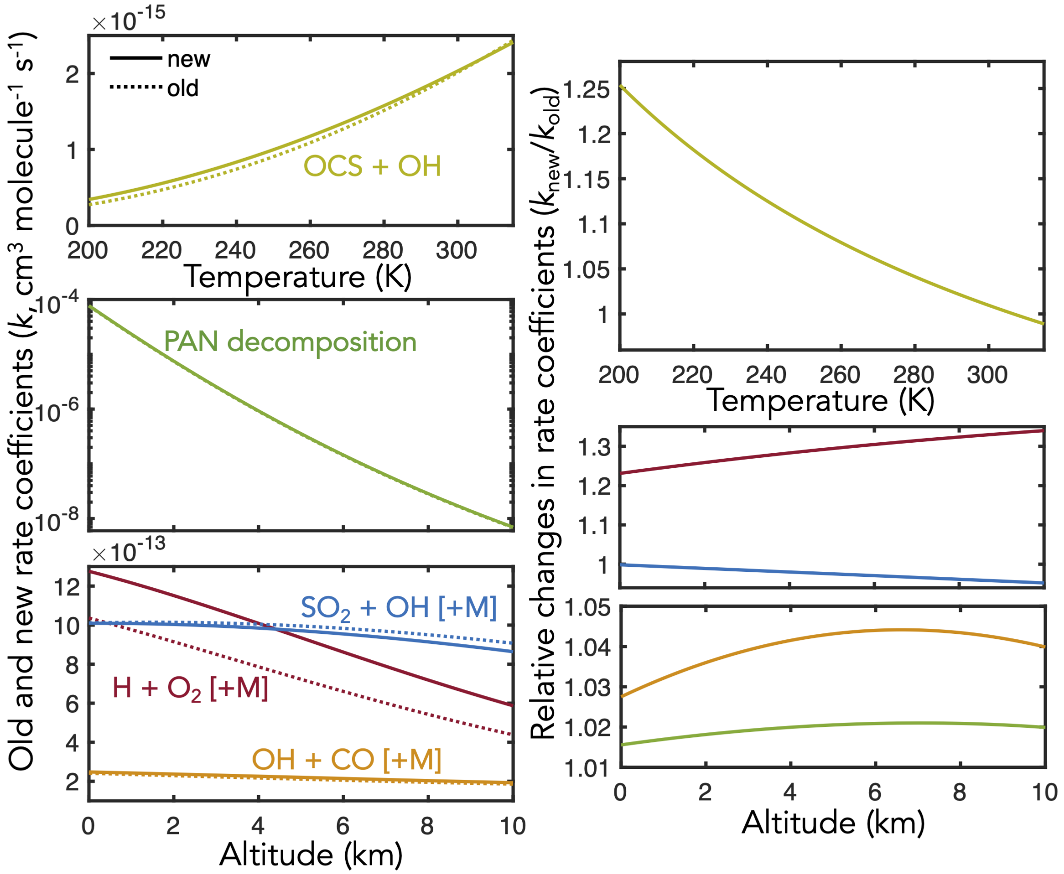

Figure S9. Base and updated rate coefficients (left) and relative difference (right) for reactions of species not in earlier categories as functions of temperature (top) and, for termolecular or decomposition reactions, altitude (bottom, where variation of temperature and pressure with altitude is shown in Figure S10).

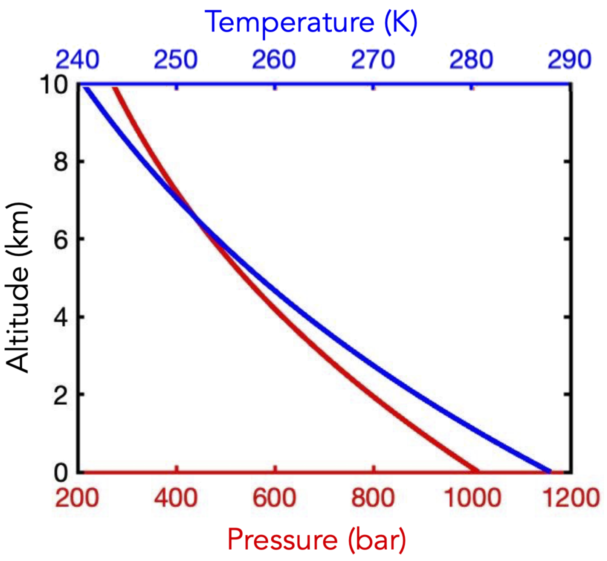

Figure S10. Parameterizations of pressure and temperature as functions of altitude for the purposes of computing rate coefficients in Figures S1 and S7-S9.

S4 GEOS-Chem updates not included in Table 1

Tables S2-S4 list existing and updated GEOS-Chem reactions mentioned in the main text but not included in Table 1. Table S2 shows the 63 reactions updated in this work to improve the balance of carbon and nitrogen between reactants and products. Table S3 shows the the 54 reactions that remain C/N imbalanced in GEOS-Chem. Both include the number of carbons or nitrogens lost or gained in the reaction, as well as the reactants and products (from the base and updated mechanism, in the case of Table 2). Table S4 shows the 23 reactions that have been simplified in this work by combining multiple branching pathways previously represented as separate reactions into a single reaction. In all three tables, lists of reaction products may be truncated for space, but any products with changed branching ratios between the old and new mechanisms are always included.

Table S2: GEOS-Chem reactions updated to improve carbon and nitrogen balance[a]

Reactants	Products (base mechanism)	C [N] imbalance[b]	Updated products
MVKOHOO + NO	0.438 MVKN	-2.248 [-0.562]	MVKN
MCO3 + MCO3	2 MO2	-2.0	2(MO2 + CO_2)
RCO3 + MCO3	MO2 + 0.5 OTHRO2 + 0.27 B3O2 + 0.07 A3O2	-1.98	2 CO_2 + MO2 + 0.49 OTHRO2 + 0.27 B3O2 + 0.07 A3O2
R4N1 + MCO3	RCHO + ACTA + NO_2	-1.5	RCHO + ACTA + NO_2 + 1.5 CO2
PIO2 + MCO3	MEK + RCHO + RCOOH + ...	-1.5	1.125 MEK + RCHO + RCOOH + CO_2 + ...
R4P + hv	OH + HO_2 + RCHO	-1.5	OH + HO_2 + 1.5 RCHO
R4O2 + MCO3	0.32(ACET + ALD2) + 0.13 RCHO + ...	-1.42	0.32(ACET + ALD2) + 0.29 RCHO + 0.94 CO_2 + ...
ICPDH + hv	0.438(HAC + GLYX) + 0.122(MCRDH + CO) + 0.133(MVKHCB + CH_2O) + 0.118(GLYC + MGLY) + ...	-1.26	0.586(HAC + GLYX) + 0.163(MCRDH + CO) + 0.1(MVKHCB + CH_2O) + 0.088(GLYC + MGLY) + ...
R4P + OH	0.791 OH + 0.791 RCHO + 0.209 R4O2	-1.1865	0.791 OH + 1.185 RCHO + 0.210 R4O2
INO2B + MCO3	CH_2O + NO_2 + MO2 + 0.903 MVK + 0.097 MACR	-1.0	CH_2O + NO_2 + MO2 + CO_2 + 0.903 MVK + 0.097 MACR
MVKHCB + OH	OH + MGLY	-1.0	OH + MGLY + CO
HMML + OH	0.7(MGLY + OH) + 0.3(MCO3 + HCOOH)	-1.0	0.7(MGLY + OH) + 0.3(MCO3 + HCOOH) + CO
MAP + HV	OH + MO2	-1.0	OH + MO2 + CO_2
RP + HV	OH + HO_2 + ALD2	-1.0	OH + HO_2 + ALD2 + CO_2
AROMRO2 + MCO3	MO2 + HO_2	-1.0	MO2 + HO_2 + CO_2
INO2D + MCO3	MO2 + 0.841 INA + 0.159(HO_2 + ICN)	-1.0	MO2 + 0.841 INA + CO_2 + 0.159(HO_2 + ICN)
MGLY	SOAGX	-1.0	1.5 SOAGX
MCO3 + OTHRO2	MO2 + HO_2 + ALD2	-1.0	MO2 + HO_2 + ALD2 + CO_2
MCO3 + A3O2	MO2 + HO_2 + RCHO	-1.0	MO2 + HO_2 + RCHO + CO_2
MCO3 + ETO2	MO2 + HO_2 + ALD2	-1.0	MO2 + HO_2 + ALD2 + CO_2
PYAC	SOAGX	-1.0	1.5 SOAGX
CH2OO + CO	CH_2O	-1.0	CH_2O + CO_2
PRN1 + MCO3	MO2 + ALD2 + CH_2O + NO_2	-1.0	MO2 + ALD2 + CH_2O + NO_2 + CO_2
B3O2 + MCO3	MO2 + HO_2 + ACET	-1.0	MO2 + HO_2 + ACET + CO_2
KO2 + MCO3	MO2 + MCO3 + ALD2	-1.0	MO2 + MCO_3 + ALD2 + CO_2
CH3CHOO + CO	ALD2	-1.0	ALD2 + CO_2
MCO3 + MO2	CH_2O + MO2 + HO_2	-1.0	CH_2O + MO2 + HO_2 + CO_2
OLNN + MCO3	MO2 + HO_2 + ...	-1.0	MO2 + HO_2 + CO_2 + ...
MCO3 + PO2	MO2 + ALD2 + CH_2O + HO_2	-1.0	MO2 + ALD2 + CH_2O + HO_2 + CO_2
ATO2 + MCO3	MO2 + MCO3 + CH_2O	-1.0	MO2 + MCO3 + CH_2O + CO_2
RCO3 + MO2	CH_2O + HO_2 + 0.5 OTHRO2 + ...	-0.98	CH_2O + CO_2 + HO_2 + 0.49 OTHRO2 + ...
RCO3 + NO	NO_2 + 0.5 OTHRO2 + 0.27 B3O2 + 0.07 A3O2	-0.98	NO_2 + CO_2 + 0.49 OTHRO2 + 0.27 B3O2 + 0.07 A3O2
KO2 + HO_2	0.85 ATOOH + 0.15(MCO3 + ALD2 + OH)	-0.85	0.85(ATOOH + MO2) + 0.15(MCO3 + ALD2 + OH)
R4O2 + MO2	0.75 CH_2O + 0.25(CH_3OH + ROH) + 0.07 RCHO + ...	-0.57	CH_2O + 0.38 ROH + 0.25 CH_3OH + 0.13 RCHO + ...
R4N1 + MCO3	MO2 + NO_2 + 0.75 ALD2 + 0.57 RCHO + ...	-0.55	MO2 + NO_2 + 0.75 ALD2 + 0.57 RCHO + 0.55 CO_2 + ...

Continued on next page

Continued from previous page

Reactants	Products (base mechanism)	C [N] imbalance[b]	Updated products
R4O2 + MCO3	MEK + ACTA	-0.5	MEK + ACTA + 0.5 CO_2
R4N1 + NO	2 NO_2 + 0.86 ALD2 + 0.57(RCHO + CH_2O)	-0.5	2 NO_2 + 0.97 ALD2 + 0.64(RCHO + CH_2O)
R4N1 + MO2	0.95 CH_2O + 0.54 RCHO + 0.38 ALD2 + 0.25 ROH + ...	-0.495	0.95 CH_2O + 0.58 RCHO + 0.38 ALD2 + 0.375 ROH + ...
RCO3 + HO_2	0.41 RP + 0.22 OTHRO2 + 0.15 RCOOH + ...	-0.43	0.43 CO_2 + 0.41 RP + 0.22 OTHRO2 + 0.15 RCOOH + ...
ATO2 + MO2	0.5(MGLY + CH_3OH + CH_2O) + 0.3 MCO3 + ...	-0.3	0.5(MGLY + CH_3OH + CH_2O) + 0.3(MCO3 + CO_2) + ...
PAN + hv	0.7(MCO3 + NO_2) + 0.3(MO2 + NO_3)	-0.3	0.7(MCO3 + NO_2) + 0.3(MO2 + NO_3 + CO_2)
KO2 + MO2	0.75 CH_2O + 0.5(ALD2 + MCO3) + ...	-0.25	0.75 CH_2O + 0.5(ALD2 + MCO3) + 0.25 MO2 + ...
R4N2 + hv	NO_2 + 0.32(ACET + ALD2 + OTHRO2) + 0.27 HO_2 + 0.19 MEK + 0.18(MO2 + B3O2) + 0.13 RCHO + ...	-0.24	NO_2 + 0.34(ACET + ALD2 + OTHRO2) + 0.27 HO_2 + 0.19 (MEK + MO2 + B3O2) + 0.15 RCHO + ...
R4O2 + NO	NO_2 + 0.32(ACET + ALD2 + OTHRO2) + 0.27 HO_2 + 0.19(MEK + MO2) + 0.18 B3O2 + 0.14 RCHO + ...	-0.2	NO_2 + 0.34(ACET + ALD2 + OTHRO2) + 0.27 HO_2 + 0.19 (MEK + MO2 + B3O2) + 0.15 RCHO + ...
PRPE + O_3	0.56 CO + 0.5 ALD2 + 0.12 CH_2OO + ...	-0.2	0.56 CO + 0.5 ALD2 + 0.22 CH_2OO + 0.1 CO_2 + ...
MVK + O_3	0.6 CH_2O + 0.545 MGLY + 0.5 CH_2OO + 0.18 CO + ...	-0.1	0.6 CH_2O + 0.545 MGLY + 0.5 CH_2OO + 0.28 CO + ...
ICNOO + NO	0.33(CO_2 + HO_2 + CO) + 0.231 PROPNN + ...	-0.099	0.429 CO_2 + 0.33(HO_2 + CO) + 0.231 PROPNN + ...
ICNOO + HO_2	0.33(CO_2 + HO_2 + CO) + 0.231 PROPNN + ...	-0.099	0.429 CO_2 + 0.33(HO_2 + CO) + 0.231 PROPNN + ...
INO2D + INO2D	0.861 ICN + 0.671 IHN1 + 0.34 INA + ...	-0.005 [-0.001]	0.862 ICN + 0.671 IHN1 + 0.34 INA + ...
HPALD2 + hv	1.637 OH + 0.818 CO + 0.455 MCRENOL + ...	+0.004	1.637 OH + 0.818 CO + 0.454 MCRENOL + ...
MACR1OOH + OH	0.488(HAC + CO) + 0.415 CO_2 + 0.165 MACR1OO + ...	+0.005	0.488(HAC + CO) + 0.41 CO_2 + 0.165 MACR1OO + ...
RCHO + hv	CO + HO_2 + 0.5 OTHRO2 + 0.27 B3O2 + 0.07 A3O2	+0.02	CO + HO_2 + 0.49 OTHRO2 + 0.27 B3O2 + 0.07 A3O2
MEK + hv	0.85 MCO3 + 0.425 OTHRO2 + 0.23 B3O2 + ...	+0.02	0.85 MCO3 + 0.415 OTHRO2 + 0.23 B3O2 + ...
KO2 + NO	0.93(NO_2 + ALD2 + MCO3) + 0.07 R4N2	+0.035	0.928 NO_2 + 0.919(ALD2 + MCO3) + 0.072 R4N2
AROMP4 + OH	0.6 GLYX + 0.45 RCOOH + 0.25 (CO + HCOOH) + ...	+0.04	0.6 GLYX + 0.43 RCOOH + 0.26 (CO + HCOOH) + ...
BENZ + OH	0.56 AROMP4 + 0.54 PHEN + 0.2 CO + 0.18 GLYX + ...	+0.04	0.55 AROMP4 + 0.54 PHEN + 0.2 CO + 0.18 GLYX + ...
IDN + hv	0.5(GLYC + HAC) + 0.228 ICN + 0.227 INA + ...	+0.05 [+0.01]	0.5(GLYC + HAC) + 0.223 ICN + 0.222 INA + ...
ITHN + OH	0.92 ITCN + 0.62 HO_2 + 0.3 OH + ...	+0.1 [+0.02]	0.90 ITCN + 0.62 HO_2 + 0.3 OH + ...
AROMP5	1.2 RCHO + 0.2(R4O2 + MGLY + HO_2)	+0.1	1.15 RCHO + 0.2(R4O2 + MGLY + HO_2) + 0.05 CO
OLND + MO2	0.965 CH_2O + 0.930 RCHO + 0.348 MEK + ...	+0.147	0.85 CH_2O + 0.930 RCHO + 0.34 MEK + ...
NPHEN + OH	AROMP4 + 0.5 R4N1 + 0.5 NO_2	+0.25	0.9 AROMP4 + 0.5 R4N1 + 0.15 CO + 0.5 NO_2
NPHEN + NO_3	AROMP4 + NO_2 + 0.5(R4N1 + HNO_3)	+0.25	NO_2 + 0.9 AROMP4 + 0.5(R4N1 + HNO_3) + 0.15 CO
HMML	SOAIE	+1.0	0.8 SOAIE

[a]See Table S1 for species names; [b]Defined as number of carbons [nitrogens in brackets] in products minus number in reactants in base mechanism (all reactions in this table are balanced in the updated mechanism).

Table S3: GEOS-Chem reactions that remain imbalanced in carbon or nitrogen[a]

C [N] imbalance[b]	Reactants	Products (base mechanism)
-7.807	LIMO2 + MCO3	0.5(RCOOH + MO2 + HO_2) + 0.385 CH_2O + 0.308 MACR + 0.192 PRPE
-7.152	LIMO2 + MO2	1.04 CH_2O + HO_2 + 0.308 MACR + 0.25(ROH + CH_3OH) + 0.192 PRPE
-7.0	HONIT + hv	HAC + NO_2
-7.0	HONIT + OH	HAC + NO_3
-7.0	MONITU + hv	RCHO + NO_2
-7.0	PIP + hv	RCHO + OH + HO_2
-6.14	PIP + OH	0.49 OH + 0.44 R4O2 + 0.41 MEK + 0.08 RCHO
-6.0	MONITS + hv	MEK + NO_2
-6.0	LIMO2 + NO_3	HO_2 + NO_2 + 0.615 MACR + 0.385(PRPE + CH_2O)
-4.726	OLND + OLND	1.21 RCHO + NO_2 + 0.7 MONITS + 0.504 CH_2O + 0.3 MONITU + 0.285 MEK
-4.52	PIO2 + NO	0.82(HO_2 + NO_2) + 0.44 MEK + 0.43 RCHO + 0.23 CH_2O + ...<br>0.12 MONITS + 0.11 ACET + 0.07 HCOOH + 0.06 MONITU
-4.137	OLND + NO_3	2 NO_2 + 1.24 RCHO + 0.464 MEK + 0.287 CH_2O
-4.137	OLND + MCO3	NO_2 + 1.24 RCHO + 0.5(RCOOH + MO2) + 0.464 MEK + 0.287 CH_2O
-4.137	OLND + NO	2 NO_2 + 1.24 RCHO + 0.464 MEK + 0.287 CH_2O
-4.0	PIO2 + MO2	HO_2 + 0.75(RCHO + MEK + CH_2O) + 0.25(ROH + CH_3OH)
-3.899	LIMO2 + NO	0.78 NO_2 + 0.686 HO_2 + 0.491 RCHO + 0.289(PRPE + ...<br>MEK) + 0.231 CH_2O + 0.22 MONITU + 0.058 HAC
-3.31	MTPO + O_3	0.85 OH + 0.65 RCHO + 0.62 KO2 + 0.53 MEK + 0.14 CO + 0.1 HO_2 + 0.02 H_2O_2
-3.31	MTPA + O_3	0.85 OH + 0.65 RCHO + 0.62 KO2 + 0.53 MEK + 0.14 CO + 0.1 HO_2 + 0.02 H_2O_2
-3.06	LIMO + O_3	0.85 OH + 0.79 MACR + 0.46 PRPE + 0.42 KO2 + 0.16 OTHRO2 + 0.14 CO + ...<br>0.1 HO_2 + 0.07 RCOOH + 0.04 CH_2O + 0.02 H_2O_2 + 0.01 HCOOH
-3.0	PIO2 + NO_3	HO_2 + NO_2 + RCHO + MEK
-2.282	OLNN + OLND	1.05 MONITS + 0.64 RCHO + 0.5(HO_2 + NO_2) + 0.45 MONITU + 0.202 CH_2O + 0.149 MEK
-2.0	OH + CH_2CCl_3	3 Cl + H_2O
-2.0	H2402 + hv	2 Br
-2.0	CH_3CCl_3 + hv	3 Cl
-1.5	O^1D + H2402	0.75(Br + BrO) + 0.25(H2402 + O)
[-1.0]	MONITU + NO_3	HONIT
[-1.0]	MONITS + NO_3	HONIT
[-1.0]	IDN	IONITA
-1.0	CH_2ICl + hv	I + Cl
-1.0	CH_2I_2 + hv	2 I
-1.0	CH_3I + hv	I
-1.0	H1211 + hv	Cl + Br
-1.0	H1301 + hv	Br
-1.0	CCl_4 + hv	4 Cl
-1.0	CH_2Cl_2 + hv	2 Cl
-1.0	CH_2Br_2 + hv	2 Br
-1.0	$CHBr_3$ + hv	3 Br
-1.0	CH_2IBr + hv	I + Br
-1.0	OH + CH_2Br_2	2 Br

Continued on next page

Continued from previous page

C [N] imbalance[b]	Reactants	Products (base mechanism)
-1.0	$OH + CHCl_3$	$3\ Cl + HO_2$
-1.0	$OH + CH_2Cl_2$	$2\ Cl + HO_2$
-1.0	$OH + CH_3Cl$	$Cl + HO_2 + H_2O$
-1.0	$OH + CH_3Br$	$Br + HO_2 + H_2O$
-1.0	$OH + CHBr_3$	3 Br
-0.95	$O^1D + CH_2Br_2$	$0.95(Br + BrO) + 0.05(CH_2Br_2 + O)$
-0.86	$O^1D + CCl_4$	$2.58\ Cl + 0.86\ ClO + 0.14(CCl_4 + O)$
-0.75	$(CH_3)_2S + OH$	$MO2 + 0.75\ SO_2 + 0.25\ CH_3SO_3H$
-0.68	$O^1D + CHBr_3$	$1.36\ Br + 0.68\ BrO + 0.32(O + CHBr_3)$
-0.64	O^1D + H1211	0.36(O + H1211) + 0.33(Br + ClO) + 0.31(BrO + Cl)
-0.41	O^1D + H1301	0.59(O + H1301) + 0.41 BrO
+0.5	R4N2	IONITA
+1.0	MCRHN	IONITA
+1.0	MCRHNB	IONITA
+1.0	MVKN	IONITA

[a]See Table S1 for species names; [b]Defined as number of carbons [nitrogens in brackets] in products minus number in reactants.

Table S4: GEOS-Chem reactions simplified by consolidating multiple branching pathways[a]

Reactants	Old reactions<br>Products	 Rate coefficient[b]	Updated reaction<br>Products	 Rate coefficient[b]
$O^1D + O_3$	$2\ O_2$<br>$2\ O + O_2$	1.2×10^{-10}<br>1.2×10^{-10}	$1.5\ O_2 + O$	2.4×10^{-10}
$O^1D + N_2O$	$N_2 + O_2$<br>$2\ NO$	$4.63 \times 10^{-11} \times e^{20/T}$<br>$7.25 \times 10^{-11} \times e^{20/T}$	$1.22\ NO + 0.39(O_2 + N_2)$	$1.19 \times 10^{-10} \times e^{20/T}$
$O^1D + CH_4$	MO2 + OH<br>$CH_2O + H + HO_2$<br>$CH_2O + H_2$	1.31×10^{-10}<br>3.5×10^{-11}<br>9.0×10^{-12}	$0.75(MO2 + OH) + 0.25\ CH_2O + 0.2(H + HO_2) + 0.05\ H_2$	1.75×10^{-10}
$H + HO_2$	2 OH<br>$H_2 + O_2$<br>$O + H_2O$	7.2×10^{-11}<br>6.9×10^{-12}<br>1.6×10^{-12}	$1.788\ OH + 0.086(H_2 + O_2) + 0.02(H_2O + O)$	8.05×10^{-11}
ClOO + Cl	$Cl_2 + O_2$<br>2 ClO	2.3×10^{-10}<br>1.2×10^{-11}	$0.95(Cl_2 + O_2) + 0.1\ ClO$	2.42×10^{-10}
IO + ClO[c]	I + OClO<br>$I + Cl + O_2$<br>$ICl + O_2$	$5.1 \times 10^{-12} \times e^{280/T}$<br>$2.81 \times 10^{-12} \times e^{280/T}$<br>$1.02 \times 10^{-12} \times e^{280/T}$	$0.809\ I + 0.56\ OClO + 0.249\ Cl + 0.191\ ICl + 0.44\ O_2$	$4.82 \times 10^{-12} \times e^{280/T}$
IO + BrO[c]	Br + OIO<br>$Br + I + O_2$	$1.2 \times 10^{-11} \times e^{510/T}$<br>$3.0 \times 10^{-12} \times e^{510/T}$	$Br + 0.8\ OIO + 0.2(I + O_2)$	$5.5 \times 10^{-12} \times e^{760/T}$
IO + IO	I + OIO<br>I_2O_2	$6.0 \times 10^{-12} \times e^{500/T}$<br>$9.0 \times 10^{-12} \times e^{500/T}$	$0.6\ I_2O_2 + 0.4(I + OIO)$	$1.5 \times 10^{-11} \times e^{500/T}$
I_2O_2 [+M]	2 IO [+M]<br>OIO + I [+M]	$1.0 \times 10^{12} \times e^{-9770/T}$<br>$2.5 \times 10^{14} \times e^{-9770/T}$	0.996(OIO + I) + 0.008 IO [+M]	$2.51 \times 10^{14} \times e^{-9770/T}$
$CH_3OOH + OH$	$CH_3OO + H_2O$<br>$CH_2O + OH + H_2O$	$2.66 \times 10^{-12} \times e^{200/T}$<br>$1.14 \times 10^{-12} \times e^{200/T}$	$H_2O + 0.7\ CH_3OO + 0.3(CH_2O + OH)$	$3.8 \times 10^{-12} \times e^{200/T}$
ATOOH + OH	$ATO2 + H_2O$<br>$MGLY + OH + H_2O$	$2.66 \times 10^{12} \times e^{200/T}$<br>$1.14 \times 10^{-12} \times e^{200/T}$	$H_2O + 0.7\ ATO2 + 0.3(MGLY + OH)$	$3.8 \times 10^{-12} \times e^{200/T}$
ETO2 + ETO2	$2(HO_2 + ALD2)$<br>EOH + ALD2	4.1×10^{14}<br>2.7×10^{-14}	$1.6\ ALD2 + 1.2\ HO_2 + 0.4\ EOH$	6.4×10^{-14}
OTHRO2 +<br>OTHRO2	$2(HO_2 + ALD2)$<br>EOH + ALD2	4.1×10^{14}<br>2.7×10^{-14}	$1.6\ ALD2 + 1.2\ HO_2 + 0.4\ EOH$	6.4×10^{-14}
RCO3 + MO2	$CH_2O + HO_2 + 0.5\ OTHRO2$<br>+ 0.27 B3O2 + 0.07 A3O2<br>$CH_2O + RCOOH$	$1.68 \times 10^{-12} \times e^{500/T}$<br><br>$1.87 \times 10^{-13} \times e^{500/T}$	$CH_2O + 0.9\ HO_2 + 0.45\ OTHRO2 +$<br>0.243 B3O2 + 0.1 RCOOH + 0.063 A3O2	$1.87 \times 10^{-12} \times e^{500/T}$
MCO3 + MO2	$CH_2O + MO2 + HO_2$<br>$CH_2O + ACTA$	$1.8 \times 10^{-12} \times e^{500/T}$<br>$2.0 \times 10^{-13} \times e^{500/T}$	$CH_2O + 0.9(MO2 + HO_2) + 0.1\ ACTA$	$2.0 \times 10^{-12} \times e^{500/T}$

Continued on next page

Continued from previous page

Reactants	Old reactions		New reaction	
	Products	Rate coefficient[b]	Products	Rate coefficient[b]
MCO3 + ETO2	ALD2 + MO2 + HO_2<br>ALD2 + ACTA	$1.68 \times 10^{-12} \times e^{500/T}$<br>$1.87 \times 10^{-13} \times e^{500/T}$	ALD2 + 0.9(MO2 + HO_2) + 0.1 ACTA	$1.87 \times 10^{-12} \times e^{500/T}$
MCO3 + OTHRO2	ALD2 + MO2 + HO_2<br>ALD2 + ACTA	$1.68 \times 10^{-12} \times e^{500/T}$<br>$1.87 \times 10^{-13} \times e^{500/T}$	ALD2 + 0.9(MO2 + HO_2) + 0.1 ACTA	$1.87 \times 10^{-12} \times e^{500/T}$
MCO3 + ATO2	MCO3 + MO2 + CH_2O<br>MGLY + ACTA	$1.68 \times 10^{-12} \times e^{500/T}$<br>$1.87 \times 10^{-13} \times e^{500/T}$	0.9(MCO3 + MO2 + CH_2O) + 0.1(MGLY + ACTA)	$1.87 \times 10^{-12} \times e^{500/T}$
MCO3 + KO2	MCO3 + MO2 + ALD2<br>MEK + ACTA	$1.68 \times 10^{-12} \times e^{500/T}$<br>$1.87 \times 10^{-13} \times e^{500/T}$	0.9(MCO3 + MO2 + ALD2) + 0.1(MEK + ACTA)	$1.87 \times 10^{-12} \times e^{500/T}$
MCO3 + PRN1	CH_2O + NO_2 + MO2 + ALD2<br>RCHO + NO_2 + ACTA	$1.68 \times 10^{-12} \times e^{500/T}$<br>$1.87 \times 10^{-13} \times e^{500/T}$	NO_2 + 0.9(CH_2O + MO2 + ALD2) + 0.1(RCHO + ACTA)	$1.87 \times 10^{-12} \times e^{500/T}$
MCO3 + A3O2	RCHO + MO2 + HO_2<br>RCHO + ACTA	$1.68 \times 10^{-12} \times e^{500/T}$<br>$1.87 \times 10^{-13} \times e^{500/T}$	RCHO + 0.9(MO2 + HO_2) + 0.1 ACTA	$1.87 \times 10^{-12} \times e^{500/T}$
MCO3 + B3O2	ACET + MO2 + HO_2<br>ACET + ACTA	$1.68 \times 10^{-12} \times e^{500/T}$<br>$1.87 \times 10^{-13} \times e^{500/T}$	ACET + 0.9(MO2 + HO_2) + 0.1 ACTA	$1.87 \times 10^{-12} \times e^{500/T}$
MCO3 + PO2	CH_2O + MO2 + ALD2 + HO_2<br>ACTA + 0.65 HAC + 0.35 RCHO	$1.68 \times 10^{-12} \times e^{500/T}$<br>$1.87 \times 10^{-13} \times e^{500/T}$	0.9(CH_2O + MO2 + ALD2 + HO_2) +<br>0.1 ACTA + 0.065 HAC + 0.035 RCHO	$1.87 \times 10^{-12} \times e^{500/T}$
MCO3 + R4O2	MO2 + 0.32(OTHRO2 + ACET<br>+ ALD2) + 0.27 HO_2 +<br>0.19 MEK + 0.18 B3O2 +<br>0.13 RCHO + 0.05 A3O2<br>MEK + ACTA	$1.68 \times 10^{-12} \times e^{500/T}$<br>$1.87 \times 10^{-13} \times e^{500/T}$	0.9 MO2 + 0.288(ACET + ALD2 + OTHRO2)<br>+ 0.271 MEK + 0.243 HO_2 + 0.162 B3O2<br>+ 0.117 RCHO + 0.1 ACTA + 0.045 A3O2	$1.87 \times 10^{-12} \times e^{500/T}$
MCO3 + R4N1	MO2 + NO_2 + 0.75 ALD2<br>+ 0.57 RCHO + 0.39 CH_2O<br>+ 0.3 R4O2<br>MEK + ACTA	$1.68 \times 10^{-12} \times e^{500/T}$<br>$1.87 \times 10^{-13} \times e^{500/T}$	NO_2 + 0.9MO2 + 0.675 ALD2 + 0.613 RCHO<br>+ 0.351 CH_2O + 0.27 R4O2 + 0.1 ACTA	$1.87 \times 10^{-12} \times e^{500/T}$

[a]See Table S1 for species names; [b]In units cm^3 $molecule^{-1}$ s^{-1} unless otherwise noted; [c]Overall rate updated per JPL; see Table 1

S5 Additional impacts of updates

Figure S11 shows changes in NO_y species between the base and updated mechanism, analogous to Figure 2 in the main text but displaying relative changes instead of absolute. Figures S12-S15 show vertical profiles of zonally averaged changes in species' annually averaged mixing ratios between the base and updated mechanism, equivalent to the profiles in Figures 1-3 and 5 of the main text but on log-pressure vertical axes. Table S4 lists the effects of the updated reactions on the annually averaged tropospheric burdens of all species that change by >1% between simulations with the old and new mechanism. Table S5 provides an analogous list for changes in stratospheric burdens, and only includes species for which >10% of the total atmospheric burden is contained in the stratosphere. Expanded tables listing the effects on every GEOS-Chem species, along with maps of the absolute and relative changes in annual average burdens of each species (without the 1% threshold) at the surface, at 500 hPa, and throughout the atmospheric column (zonally averaged), can be found on the Harvard data repository (DOI 10.7910/DVN/IDYV3E).

Table S5: Changes in annual average tropospheric burdens from the base mechanism to the updated mechanism.[a]

Species name	Tropospheric burden (Gg)[b]	Absolute change (Gg)	Relative change (%)
A3O2	0.0987	-0.00194	-1.93
ACET	5520	188	3.54
ACTA	348	-12.1	-3.37
ALD2	305	3.38	1.12
AROMP4	0.166	-0.00204	-1.22
AROMRO2	0.127	-0.00145	-1.13
B3O2	0.417	0.0492	13.4
BENZO	0.00969	-0.000127	-1.30
BENZO2	0.346	-0.00365	-1.04
BrNO2	0.0977	-0.00287	-2.86
BrNO3	5.80	-0.0886	-1.51
BRO2	0.0761	-0.000832	-1.08
C2H4	121	-2.17	-1.77
C3H8	494	-97.5	-16.5
C4HVP1	0.00187	-0.000063	-3.24
C4HVP2	0.00530	-0.000056	-1.05
CH2OO	0.000005	0.000002	86.9
CH3CHOO	0.000009	-0.000001	-8.38
Cl	0.00014	0.000002	1.36
Cl2	1.04	0.0198	1.94
Cl2O2	0.000516	0.000111	27.4
ClO	0.299	0.0119	4.14
ClOO	0.000014	<1E-6	3.02
CO	286000	-8080	-2.75
CO2	159	6.68	4.38

Continued on next page

Continued from previous page

Species name	Tropospheric burden (Gg)[b]	Absolute change (Gg)	Relative change (%)
ETHLN	0.809	0.0573	7.63
ETHN	2.48	-0.0379	-1.51
ETHP	41.2	-0.660	-1.58
ETO	<1E-6	<1E-6	-1.11
ETOO	0.330	-0.0124	-3.62
ETP	86.4	5.73	7.10
H	<1E-6	<1E-6	-21.5
H2O2	2750	34.6	1.28
HCOOH	554	16.7	3.10
HMHP	31.4	-8.67	-21.7
HMML	12.3	-0.242	-1.94
HNO2	2.93	-0.0375	-1.27
HNO4	67.5	1.52	2.30
HPALD1	2.60	-0.132	-4.84
HPALD1O	0.00554	-0.000509	-8.42
HPALD2	7.40	-0.0957	-1.28
HPALD2O	0.0169	-0.000765	-4.32
HPALD3	2.14	-0.0901	-4.04
HPALD4	5.08	-0.0551	-1.07
HPETHNL	3.78	-0.119	-3.06
I	0.819	-0.0122	-1.47
I2O3	0.114	-0.00180	-1.55
I2O4	0.00216	-0.000133	-5.79
ICHOO	0.00120	0.000019	1.63
ICPDH	4.44	0.0777	1.78
IDHDP	10.9	0.247	2.33
IDHNBOO	0.992	-0.0187	-1.86
IDHNDOO	0.00207	0.000055	2.71
IDHNDOO	0.00150	0.000017	1.15
IEPOXAO	0.00931	0.000309	3.44
IEPOXBO	0.00371	0.000124	3.45
IHN1	1.84	0.0260	1.44
IHN4	0.426	0.00423	1.00
IHOO1	1.02	-0.0241	-2.30
IHPOO2	0.00336	0.000059	1.80
IHPOO3	0.0214	0.000273	1.29
IHPNBOO	0.00182	0.000019	1.06
INA	<1E-6	<1E-6	1.56
INO	0.00254	-0.000161	-5.96
INO2B	1.08	0.0126	1.18
INPB	3.86	0.0509	1.34
INPD	4.23	0.0446	1.07
IONO	0.136	-0.0138	-9.17

Continued on next page

Continued from previous page

Species name	Tropospheric burden (Gg)[b]	Absolute change (Gg)	Relative change (%)
IONO2	2.14	-0.0279	-1.28
IPRNO3	51.8	4.09	8.57
ISOPNOO	0.00554	0.000125	2.30
ISOPNOO	0.00414	0.000062	1.51
ITHN	10.0	0.133	1.34
KO2	1.07	-0.0159	-1.47
LIMO2	0.0287	-0.00228	-7.34
LVOC	0.0967	0.00170	1.79
LVOCOA	14.4	0.228	1.60
MACR	63.4	-1.39	-2.15
MACR1OO	0.0594	-0.00198	-3.23
MACR1OO	5.22	-0.0822	-1.55
MAP	670	194	40.9
MCRDH	10.1	0.107	1.07
MCO3	1.39	-0.0632	-4.34
MCROHOO	0.00671	-0.000247	-3.55
MEK	367	9.71	2.72
MO2	24.9	0.280	1.14
MP	2320	28.7	1.25
MPAN	7.09	-0.132	-1.82
MVKDH	54.0	0.608	1.14
MVKOHOO	0.603	-0.0126	-2.05
MVKN	2.73	0.774	39.4
N	<1E-6	<1E-6	-48.9
N2O5	25.5	-0.393	-1.52
NO	58.0	-1.42	-2.38
NO2	291	-3.03	-1.03
NPRNO3	10.8	-0.929	-7.94
OIO	0.152	-0.00327	-2.10
OLND	0.547	-0.0146	-2.59
OLNN	0.0701	-0.00248	-3.42
OTHRO2	0.599	0.0574	10.6
PAN	1430	-56.5	-3.81
PIO2	0.393	-0.0221	-5.32
PO2	0.218	-0.0115	-5.01
PP	26.6	-0.533	-1.96
PPN	292	24.2	9.04
PRN1	0.250	-0.00928	-3.58
PRPE	35.0	-0.854	-2.38
RA3P	17.4	-0.781	-4.29
RB3P	47.6	5.34	12.6
RCHO	69.6	4.78	7.37
RCO3	0.243	0.0246	11.2

Continued on next page

Continued from previous page

Species name	Tropospheric burden (Gg)[b]	Absolute change (Gg)	Relative change (%)
ROH	5.03	0.877	21.1
RP	62.1	7.53	13.8
TRO2	0.0733	-0.00131	-1.76
XRO2	0.0562	-0.00105	-1.82

[a]Only species with changes of >1% are listed; see Table S1 for species names; [b]In a simulation with the updated mechanism.

Table S6: Changes in annual average stratospheric burdens from the base mechanism to the updated mechanism.[a]

Species name	Stratospheric burden (Gg)[b]	% of total burden in stratosphere[b]	Absolute change (Gg)	Relative change (%)
BrO	8.12	80	-0.104	-1.26
BrSALA	0.0841	27	0.00752	9.81
CHCl3	19.9	12	-1.24	-5.88
Cl2	2.62	72	0.0338	1.30
ClO	42.6	99	-1.76	-3.97
ClOO	0.000512	97	0.000007	1.39
H	0.000301	>99	-0.000076	-20.2
HBr	0.826	16	0.0512	6.60
HCl	681	76	6.96	1.03
HNO2	1.19	29	0.0286	2.46
HNO3	4880	79	75.6	1.57
HO2	5.99	19	-0.0790	-1.30
HOBr	4.20	52	-0.0475	-1.12
HOCl	24.8	90	-1.49	-5.67
I	0.261	24	-0.00862	-3.19
I2O2	0.0172	48	0.000484	2.89
INO	0.0009	26	-0.000134	-13.0
IONO	0.125	48	-0.0140	-10.1
N	0.000092	>99	-0.000012	-11.5
N2O5	523	95	33.5	6.84
NIT	45.3	10	0.951	2.15
NO	524	90	38.4	7.91
NO2	1510	84	99.2	7.03
O1D	0.000026	>99	-0.000001	-3.70
OClO	3.25	98	-0.168	-4.91
OIO	0.0525	26	0.00464	9.69
PPN	74.3	20	4.76	6.84

[a]Only species with changes of >1% *and* for which >10% of the total burden is contained in the stratosphere are listed; see Table S1 for species names; [b]In a simulation with the updated mechanism.

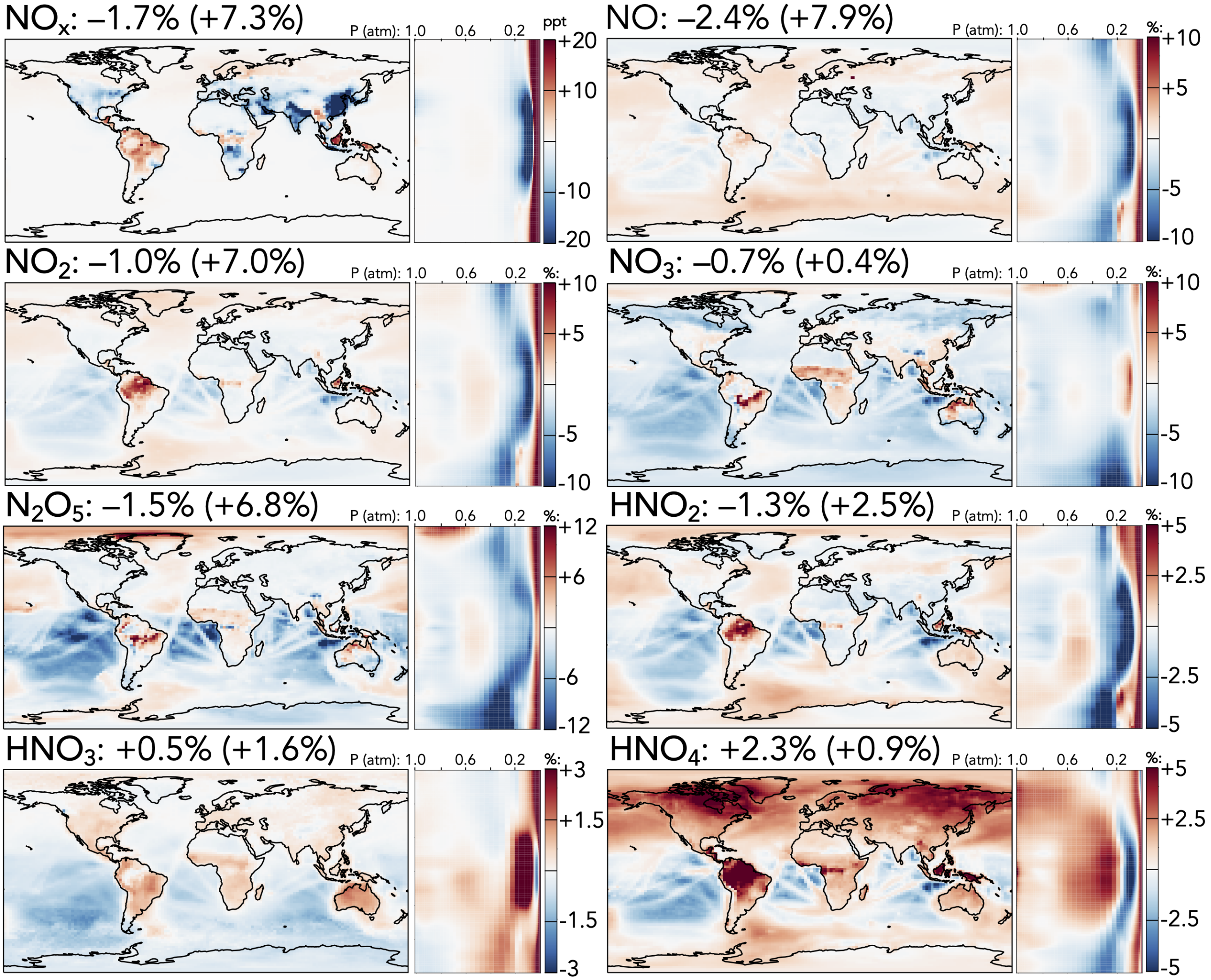

Figure S11. Relative (top left, for NO_x) and absolute (all others) changes in the annual average mixing ratios of NO_z species between the base and updated mechanism. Maps show surface values; atmospheric cross-sections show zonal means using the labelled altitude scale and the same latitude scales as the maps to their left. Scales differ between species but are the same for each individual species' surface maps and cross-sections. Numbers next to species names show the percent change in their annual average tropospheric burden (stratospheric burden in parentheses) from the base to the updated mechanism.

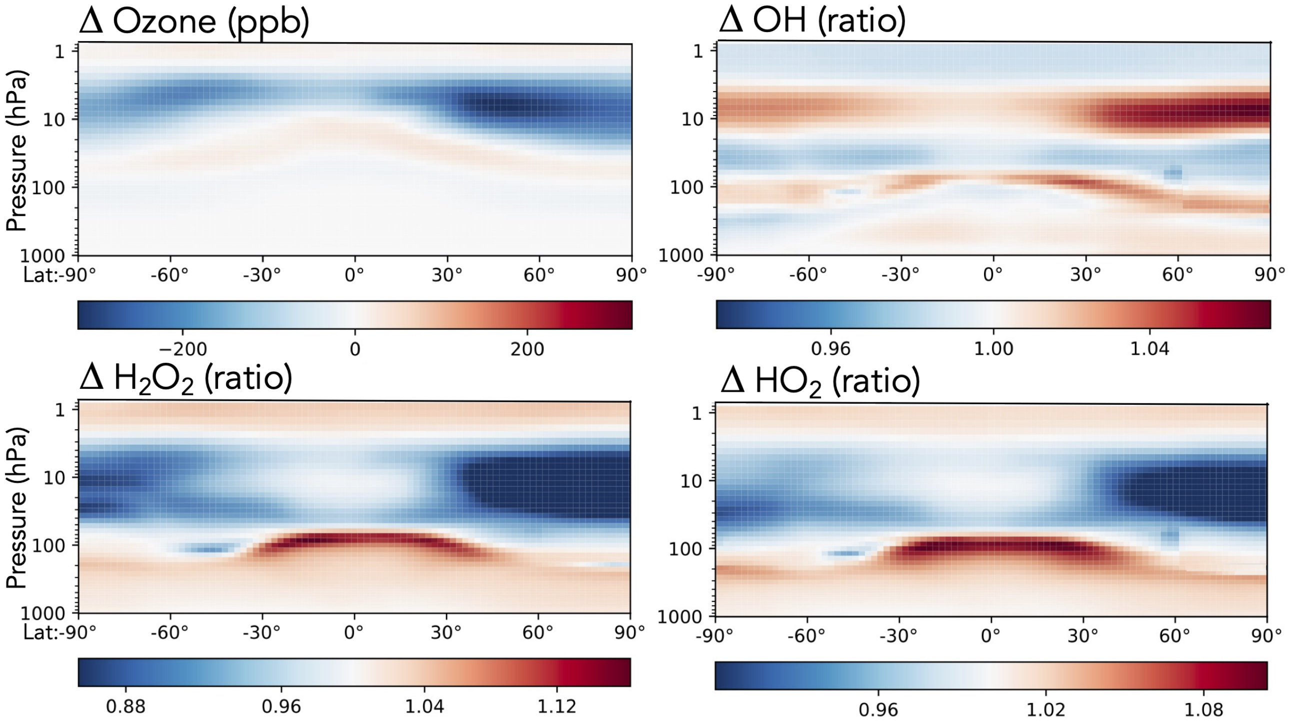

Figure S12. Absolute (top left, for ozone) and relative (all others) changes in the vertical profiles of zonal mean annual average mixing ratios of (H)O_y species between the base and updated mechanism. Scales differ between species and, for relative scales, show to the value of [(updated – base) / base] mixing ratios. Plotted values are equivalent to those in the zonal profiles of Figure 1 in the main text, but on log-pressure axes.

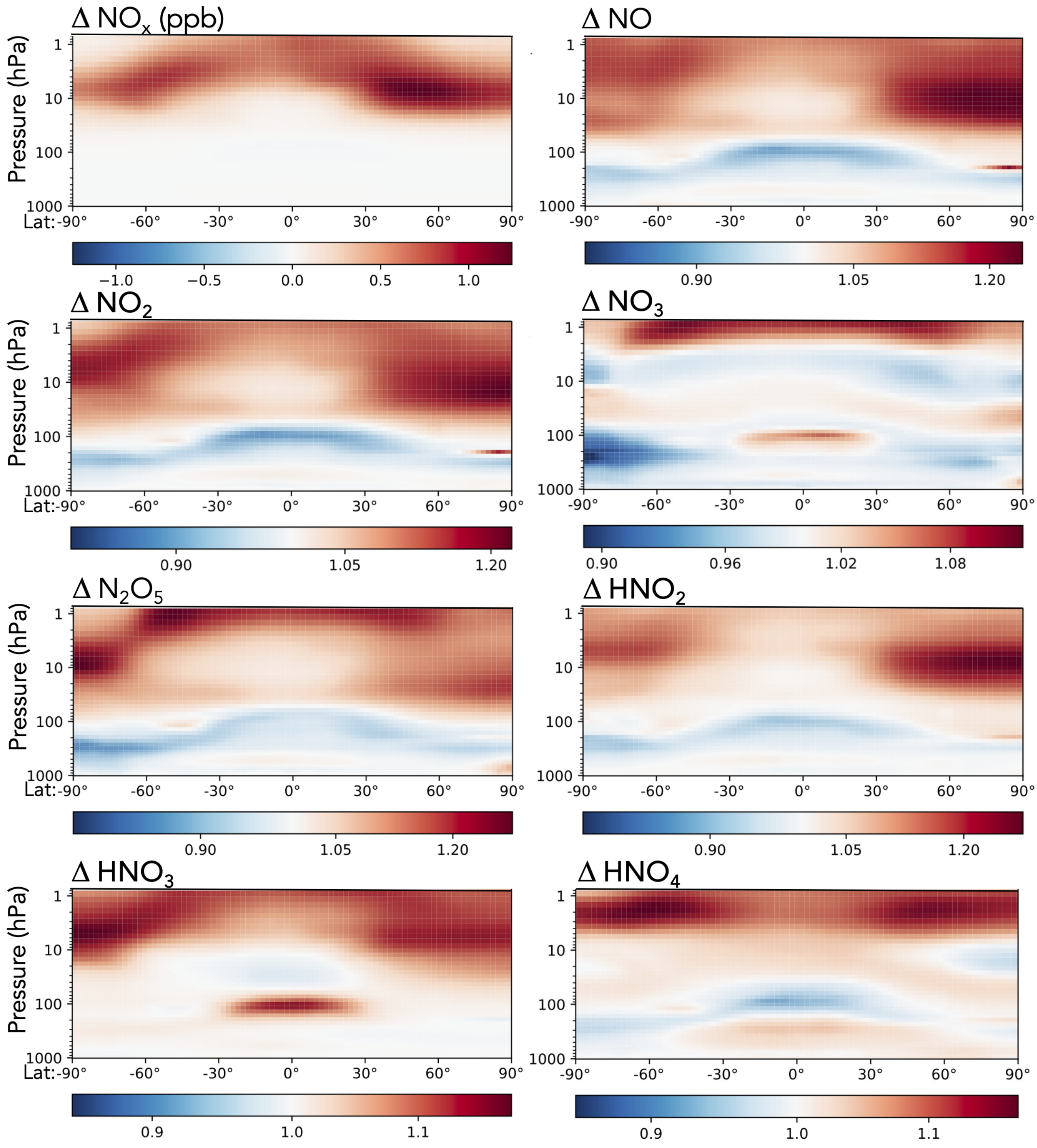

Figure S13. Absolute (top left, for ozone) and relative (all others) changes in the vertical profiles of zonal mean annual average mixing ratios of NO_y species between the base and updated mechanism. Scales differ between species and, for relative scales, show to the value of [(updated – base) / base] mixing ratios. Plotted values are equivalent to those in the zonal profiles of Figure S11, but on log-pressure axes.

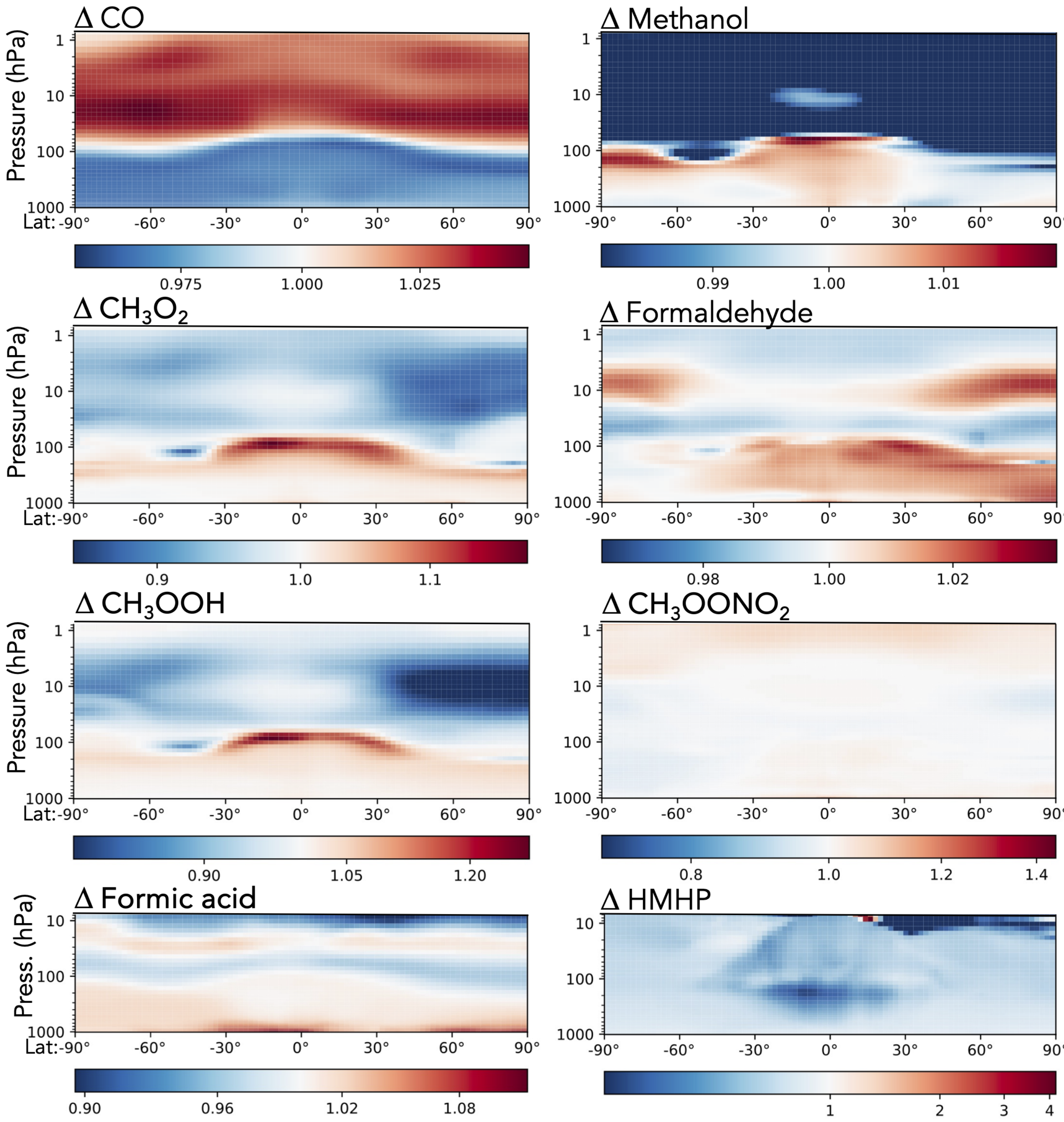

Figure S14. Relative changes in the vertical profiles of zonal mean annual average mixing ratios of C_1 species between the base and updated mechanism. Scales differ between species and show to the value of [(updated – base) / base] mixing ratios. Plotted values are equivalent to those in the zonal profiles of Figure 3 in the main text, but on log-pressure axes.

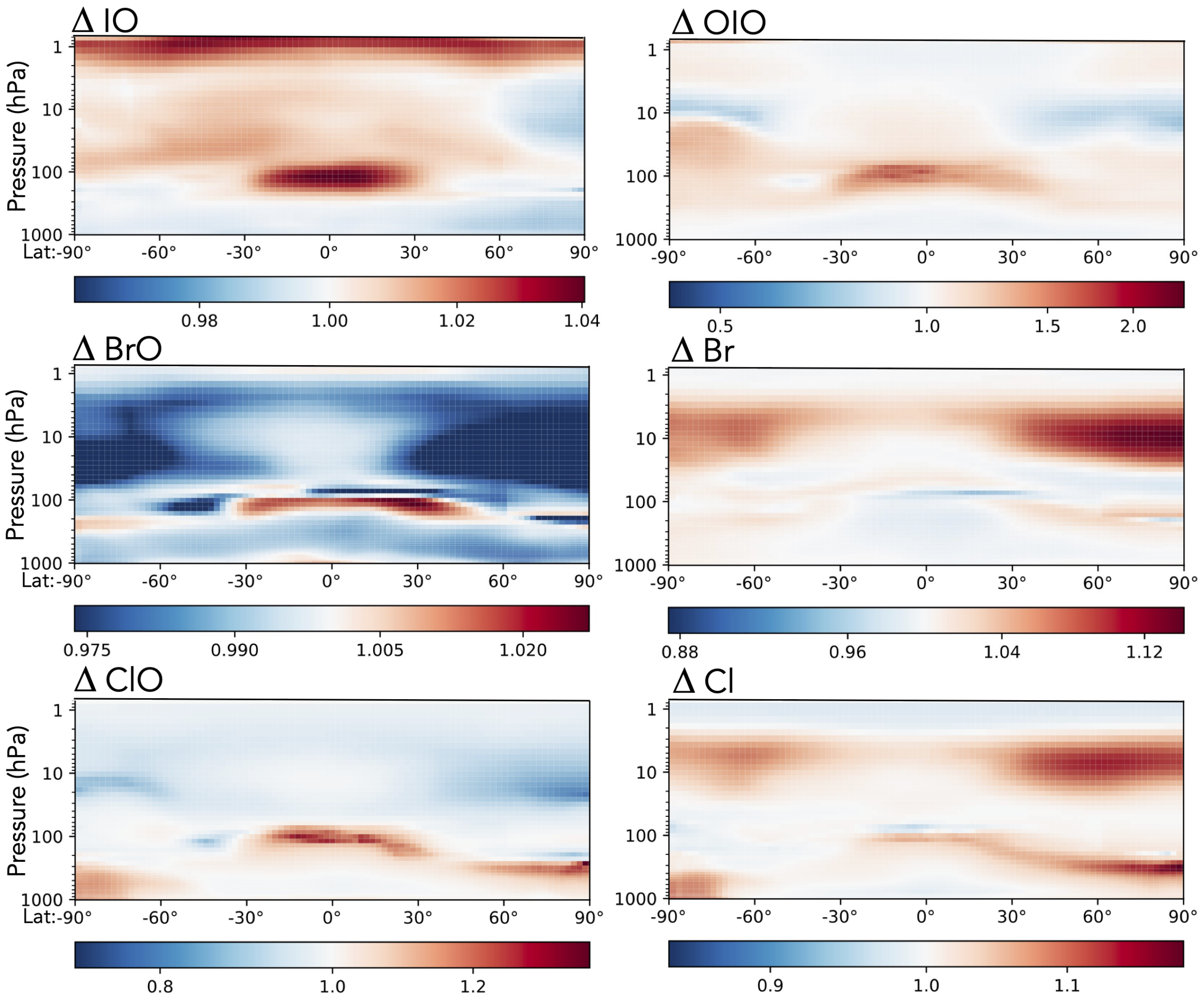

Figure S15. Relative changes in the vertical profiles of zonal mean annual average mixing ratios of select halogen species between the base and updated mechanism. Scales differ between species and show to the value of [(updated – base) / base] mixing ratios. Plotted values are equivalent to those in the zonal profiles of Figure 5 in the main text, but on log-pressure axes.
